# Changes in five cerebrospinal fluid and three plasma Alzheimer’s disease biomarkers across increasing brain amyloid-beta and tau pathology burden

**DOI:** 10.1007/s00401-026-03076-5

**Published:** 2026-09-03

**Authors:** Andrea Mastrangelo, Simone Baiardi, Edoardo Ruggeri, Giuseppe Mario Bentivenga, Claudia Marina Vargiu, Angela Mammana, Marco Sbriccoli, Barbara Polischi, Benedetta Carlà, Sabina Capellari, Piero Parchi

**Affiliations:** 1 Department of Biomedical and Neuromotor Sciences, University of Bologna, Bologna, Italy; 2 IRCCS, Istituto Delle Scienze Neurologiche di Bologna, Bologna, Italy; 3 Department of Neuroscience, Istituto Superiore di Sanità, Rome, Italy

**Keywords:** Autopsy, Neuropathology, Diagnosis, Staging, Validation, Neurodegenerative diseases

## Abstract

Neuropathological validation studies of pathology-specific biomarkers for neurodegenerative diseases are essential but are often limited by long sampling-to-death intervals and the lack of semi-quantitative pathology measures. We assessed the associations of five cerebrospinal fluid (CSF) (Aβ_42_/Aβ_40_, p-tau_181_, p-tau_217_, Aβ_42_/p-tau_181_ and Aβ_42_/p-tau_217_) and three plasma (p-tau_217_, p-tau_217_/Aβ_42_ and Aβ_42_/Aβ_40_) biomarkers with post-mortem Aβ and tau pathology burden in 250 participants with ante-mortem CSF (*n=*230) and/or plasma (*n=*101), affected by prion (*n=*162) or non-prion diseases (*n=*88). Aβ and tau burden were scored across nine and six brain areas, respectively. We assessed the earliest biomarker changes across quartiles of Aβ and tau pathology burden and the discriminatory performance at progressively higher pathology thresholds using multivariable linear regression and sequential ROC analyses. Analyses on p-tau markers were restricted to non-prion participants. The median sampling-death interval was 1.5 months for CSF and 1 month for plasma. CSF Aβ_42_/Aβ_40_ decreased at the second quartile of Aβ burden (*p<*0.001), whereas p-tau_181_ (*p<*0.01) and p-tau_217_ (*p<*0.001) increased only from the third quartile. CSF Aβ_42_/Aβ_40_ achieved its highest accuracy at low/intermediate Aβ burden (AUC 0.984), while CSF p-tau and derived ratios performed best at advanced Aβ (AUCs 0.889 to 0.980) and intermediate tau pathology stages (AUCs 0.949 to 0.995). CSF p-tau_217_ and Aβ_42_/p-tau_217_ consistently showed higher accuracy than their p-tau_181_ counterparts across Aβ and tau pathology scores. Plasma p-tau_217_ and p-tau_217_/Aβ_42_ significantly increased in the highest Aβ and tau burden quartiles, where they achieved their best performance (AUCs 0.893 to 0.928). These findings support a sequential model of biomarker changes across the Alzheimer’s disease neuropathological continuum. CSF Aβ_42_/Aβ_40_ best reflects low/intermediate Aβ burden, while CSF p-tau markers are more closely related to high Aβ and intermediate tau load. Plasma markers primarily identify advanced Aβ and tau pathology burden. Notably, current fluid biomarkers do not capture the earliest phases of Aβ deposition.

**Supplementary Information:**

The online version contains supplementary material available at https://doi.org/10.1007/s00401-026-03076-5.

## Introduction

Alzheimer’s disease (AD) is neuropathologically characterized by the accumulation of misfolded amyloid-β (Aβ) and hyperphosphorylated tau (p-tau) proteins in the form of amyloid plaques and neurofibrillary degeneration. Alterations in Aβ processing and clearance are generally considered the initiating events in the AD pathogenic cascade, leading to the subsequent accumulation of toxic p-tau aggregates [16, 31, 53].

The identification of fluid biomarkers reflecting the pathological AD hallmarks has shifted AD diagnosis toward a biological definition [18]. Specifically, cerebrospinal fluid (CSF) levels of Aβ_42_/Aβ_40_ peptides strongly correlate with post-mortem Aβ plaque burden and show high accuracy in identifying subjects with a neuropathological diagnosis of AD [8, 19, 34]. Similarly, the quantification of different isoforms of soluble p-tau (e.g., p-tau_181_, p-tau_231_, p-tau_217_) in CSF and, more recently, in blood has provided additional validated biomarkers of AD pathology [4–6, 21, 22, 30, 34, 40]. Among these, p-tau_217_ has shown the highest accuracy in identifying subjects with AD, even at the preclinical stage, and the widest fold-changes across the AD continuum [6, 40]. Current diagnostic criteria incorporate these biomarkers, expanding the diagnostic window to prodromal and preclinical stages [18]. Accordingly, most studies on early diagnosis along the AD continuum focus on clinical rather than pathological stages [6, 25, 35, 37]. However, this approach has limitations, as progression across clinical stages does not necessarily parallel increases in AD pathology burden, because resilience mechanisms and co-pathologies may shape the clinical phenotype. Validation studies against neuropathology are therefore essential to determine which level of AD pathological burden corresponds to specific biomarker alterations. Anchoring biomarker alterations to pathological rather than clinical stage may also have therapeutic implications, as preliminary evidence suggests greater clinical efficacy of anti-amyloid antibodies at earlier AD pathological stages, as predicted by tau-PET [49, 57]. Moreover, amyloid plaque clearance seems to occur more rapidly in subjects with lower Aβ burden [48].

Available studies indicate that CSF Aβ_42_/Aβ_40_ decreases nonlinearly from early Thal Aβ phases [8, 34]. Changes in soluble p-tau isoform levels, including p-tau_181_ and p-tau_217_ in both CSF and blood, appear to occur at later stages, although results remain inconsistent [15, 27, 28, 34, 58, 59]. Furthermore, the relationship between the earliest biofluid alterations and semi-quantitative Aβ and tau burden, independent of categorical disease stages, remains largely unexplored. Notably, categorical staging systems (such as Thal phases and Braak stages) do not fully reflect the actual regional pathological burden. Moreover, most available neuropathological studies are limited by long time intervals between CSF/blood sampling and neuropathological assessment. Under these conditions, a possible discrepancy in AD stages between the two time points cannot be completely ruled out, and its impact is difficult to estimate.

Here, we studied a large neuropathology autopsy cohort with matched CSF and/or plasma samples taken shortly before death (around one month for both CSF and plasma). In all cases, we semi-quantitatively scored Aβ and tau pathology severity across nine and six brain regions, respectively. We investigated associations between five CSF (Aβ_42_/Aβ_40_, p-tau_181_, p-tau_217_, Aβ_42_/p-tau_181_, and Aβ_42_/p-tau_217_) and three plasma (p-tau_217_, p-tau_217_/Aβ_42_, and Aβ_42_/Aβ_40_) markers and semi-quantitative severity scores of Aβ and tau proteinopathies, and determined the earliest pathological burden associated with significant alterations in their levels. We also assessed the discriminatory performance of different biomarkers against Aβ and tau pathologies at increasing levels of pathology burden.

## Methods

### Participant selection and cohort definition

We studied subjects with available post-mortem brain tissue submitted to the Neuropathology Laboratory (NP-Lab) at the Institute of Neurological Sciences in Bologna between January 2003 and January 2026. The inclusion criteria for the present study were as follows: 1) availability of at least one antemortem CSF (CSF cohort) and/or plasma (plasma cohort) sample; 2) sufficient CSF/plasma to perform biomarker analyses (see below); 3) sufficient and qualitatively adequate brain tissue to perform AD neuropathological diagnosis and staging. We included 250 participants (162 (64.8%) with a final diagnosis of prion disease) in the final cohorts (CSF cohort, *n=*230; plasma cohort, *n=*101).

Post-mortem neuropathological examination was performed at the NP-Lab in 245 out of 250 cases (98.0%). In most participants (237/250, 94.8%), an autopsy was performed within the Italian Surveillance Program for prion disease following clinical suspicion of prion disease due to a rapidly progressive neurological syndrome. In the remaining cases (13/250, 5.2%), neuropathological examination was carried out to achieve a definite diagnosis in subjects with slowly progressive neurological disease of a suspected degenerative origin.

### Neuropathological examination

Neuropathological examination was performed according to a standardized protocol, as previously described [8, 44]. Each brain was divided sagittally; the left hemibrain was fixed in 10% buffered formalin, while the right was sectioned coronally and immediately frozen at − 80 °C in sealed plastic bags. The formalin-fixed hemibrain was serially sectioned in 1 cm-thick slices, and tissue blocks from 23 brain regions were processed routinely to obtain paraffin-embedded tissue blocks.

Seven-μm-thick sections from each block were initially stained with hematoxylin–eosin for screening. In selected brain areas of all cases, immunohistochemistry was performed with antibodies specific for PrP (3F4, dilution 1:400, BioLegend), Aβ (4G8, dilution 1:5000, BioLegend), phospho-tau (p-tau) (AT8, dilution 1:200, Invitrogen) and α-synuclein (LB509, dilution 1:100, Invitrogen), according to established consensus criteria [1, 2, 7, 42]. Further antibodies were employed in selected cases, following clinical suspicion and/or findings on hematoxylin–eosin staining (hyperphosphorylated TDP-43: phospho Ser 409/410–1 polyclonal antibody, 1:5000, CosmoBio Co; GFAP: 6F2, 1:100, Agilent Dako; CD3: SP7, 1:200, Diagnostic BioSystems; CD8: CD8/144B, 1:100, Dako Cytomation). In all cases, an experienced neuropathologist (P.P.) formulated the final neuropathological diagnosis.

### Assessment of Alzheimer’s disease pathology

In all included participants, an experienced neuropathologist staged both amyloid and neurofibrillary pathologies according to Thal phases [51] and Braak stages [12]. Furthermore, a CERAD neuritic plaque score was assigned to each subject. All participants were classified according to the level of AD neuropathologic change (ABC score) [38].

A semi-quantitative scoring of both amyloid and tau pathologies was performed in all cases, as previously described [8], independently by two evaluators (P.P. and either A.Mas. or S.B.), blinded to biomarker results.

For Aβ burden, the following nine brain areas were evaluated: cerebral cortex (frontal, parietal, temporal and occipital lobes), amygdala, hippocampus (CA1 region), striatum, midbrain (periaqueductal area) and cerebellum. For each region, parenchymal Aβ and cerebral amyloid angiopathy (CAA) pathologies were scored separately. For parenchymal deposits, the density of amyloid plaques was graded as follows: 0) entirely negative; 2) rare or sparse deposits (2–4 plaques in at least one 100× microscopic field); 4) moderate number of deposits (5–10 plaques); 6) multiple and disseminated deposits (> 10 plaques). An additional point was added for each area if core plaques were detected. In each area, CAA pathology was graded as follows: 0) no vessels involved; 1) up to two vessels focally involved; 2) intermediate between 1 and 3; 3) more than half of the vessels involved. A cumulative Aβ score (0–90) was then calculated for each participant.

As for AD-related tau pathology, the densities of neuropil threads, neurofibrillary tangles and thick neurites were separately scored (0–3) in six brain regions: the transentorhinal cortex, entorhinal cortex, parahippocampal gyrus, middle temporal gyrus, middle frontal gyrus and occipital cortex. The following scoring system was adopted: 0) entirely negative; 1) mild immunoreactivity; 2) moderate immunoreactivity; 3) severe immunoreactivity. A cumulative tau score (0–54) was attributed to each participant.

### Cerebrospinal fluid and plasma biomarker analyses

Antemortem CSF samples were obtained by lumbar puncture at the L3–L4 or L4–L5 interspinal space following a standard procedure, centrifuged at 1000 g for 15 min, divided into aliquots and stored at − 80 °C until analysis. EDTA plasma samples were collected, aliquoted and stored at −80 °C according to standard procedures.

CSF p-tau_181_, p-tau_217_, Aβ_42_ and Aβ_40_ and plasma p-tau_217_, Aβ_42_ and Aβ_40_ levels were measured by automated chemiluminescent enzyme immunoassays (CLEIA) on the Lumipulse G1200 platform (Fujirebio, Gent, Belgium). The coefficients of variation were below 8% for all analyses.

In detail, CSF/plasma Aβ_42_ and Aβ_40_ were measured in the entire CSF and plasma cohorts, including participants with prion disease. Conversely, analyses involving tau-related biomarkers (CSF p-tau_181_, CSF p-tau_217_, plasma p-tau_217_ and derived ratios) were restricted to non-prion participants due to the reported direct effect of prion disease pathology on tau biomarker levels, including phosphorylated species [10]. Finally, CSF p-tau_217_ measurements were available only in a subset of the non-prion cohort with sufficient residual CSF for analysis (*n=*61), as this biomarker was assayed later than the others.

### Genetic analyses

APOE genotyping was performed in all participants using restriction fragment length polymorphism, as previously described [60].

### Statistical analyses

The analyses were performed using R version 4.4.3 (R Foundation for Statistical Computing, Vienna, Austria) and GraphPad Prism version 7 (GraphPad, La Jolla, CA, USA). Because biomarker values were not normally distributed (Shapiro–Wilk and Kolmogorov–Smirnov tests), values were log-transformed for analyses.

The independent associations between continuous Aβ and tau scores (independent variables) and log-transformed biomarker values (dependent variables) were assessed using multivariable linear regression models with the following predictors: 1) only continuous Aβ pathology score; 2) only continuous tau pathology score; and 3) both Aβ and tau pathology scores. Age at CSF/plasma sampling, sex, time from CSF/plasma sampling to death and prion disease status (when applicable) were included as covariates.

The earliest significant biomarker changes in relation to semi-quantitative Aβ and tau pathology burden were evaluated using multivariable linear regression models with categorical predictors. In detail, Aβ and tau scores greater than zero were categorized into quartiles, separately within each analysis subgroup (i.e., entire cohort and non-prion participants). Multivariable linear regression models were fitted, including categorical Aβ or tau score levels as independent variables and log-transformed biomarker values as dependent variables. Participants with a score of zero were used as the reference group. All models were adjusted for age at CSF/plasma sampling, sex, time between CSF/plasma sampling and death and prion disease status (when applicable). Sensitivity analyses for CSF biomarkers were performed in the subgroup of participants with complete CSF biomarker availability. When assessing associations with tau pathology, further sensitivity analyses were performed by 1) excluding subjects with tau pathology in the absence of Aβ deposition (i.e., primary age-related tauopathy (PART)) and 2) additionally adjusting for Aβ score. Secondary multivariable linear regression models were fitted to evaluate the association between categorical standard neuropathological scores (i.e., Thal phase, Braak stage and ADNC levels) (independent variables) and log-transformed biomarker values (dependent variables).

To assess the discriminatory performance of biomarkers across increasing levels of Aβ and tau burden, sequential receiver operating characteristic (ROC) analyses were performed using progressively higher thresholds of Aβ and tau scores. For each curve, subjects with a pathology score at or below the threshold were deemed “negative”, while those with values above the threshold were classified as “positive”. Thresholds were defined using fixed intervals to reflect the different ranges of Aβ and tau scores while ensuring a comparable number of analyses across Aβ and tau pathologies. Specifically, Aβ scores and tau scores were grouped into 8-unit and 5-unit intervals, respectively. Subjects with a score of zero were included in the lowest interval, whereas subjects with values above the 90th percentile of the entire cohort were grouped in the highest interval. For each pathology threshold, two biomarker cut-offs were determined: one based on the maximized Youden index and another selected to achieve an approximately 95% specificity. Sensitivity analyses were performed in a subgroup of participants for whom results for all examined biomarkers were available. Secondary ROC curves were performed to assess discrimination across different levels of standard neuropathological scoring systems. All analyses were two-sided and considered significant at *p<*0.05.

## Results

### Demographic and neuropathological features of the included participants

The demographic features of included participants, stratified by CSF and plasma sample availability and prion disease status, are reported in Table 1. A total of 230 participants had a CSF sample available (CSF cohort). Of these, 71 (30.9%) belonged to the non-prion CSF cohort. Plasma samples were available in 101 participants (plasma cohort), 60 of whom (59.4%) had a non-prion disease (non-prion plasma cohort). The median age at death was around 70 years in all subgroups, and the median time between CSF sampling and death was around 1 month (CSF cohort: 1.5, interquartile range (IQR) 0.5–4.7; non-prion CSF cohort: 1.2, IQR 0.3–7.7). Similar values were obtained for plasma samples (plasma cohort: 1.0, IQR 0.3–4.4; non-prion plasma cohort: 0.9, IQR 0.2–8.5).

**Table 1:** Demographic and basic neuropathological features in the entire cohort and subgroups

	Entire cohort (*N=*250)	CSF cohort (*N=*230)	Non-prion CSF cohort (*N=*71)	Plasma cohort (*N*=101)	Non-prion plasma cohort (*N*=60)
CSF available	230 (92.0)	230 (100)	71 (100)	81 (80.2)	43 (71.7)
Plasma available	101 (40.4)	81 (35.2)	43 (60.6)	101 (100)	60 (100)
Age at CSF sampling^a^	70.3 (63.0–77.8)	70.3 (63.0–77.8)	74.1 (63.1–80.1)	72.0 (64.5–78.5)	74.2 (63.1–80.6)
Age at plasma sampling^a^	72.3 (65.0–78.1)	72.0 (64.5–78.5)	74.2 (65.7–80.6)	72.3 (65.0–78.1)	73.1 (65.8–78.9)
Age at death^a^	72.0 (63.7–77.6)	70.7 (63.6–77.8)	74.6 (65.8–80.2)	73.2 (65.8–78.2)	74.7 (67.5–79.6)
Females	122 (48.8)	114 (49.6)	40 (56.3)	52 (51.5)	31 (51.7)
*APOE* ε4 carriers	43 (17.2)	38 (16.5)	13 (18.3)	22 (21.8)	15 (25.0)
Time CSF sampling-death^b^	1.5 (0.5–4.7)	1.5 (0.5–4.7)	1.2 (0.3–7.7)	1.0 (0.4–3.3)	0.9 (0.2–5.4)
Time plasma sampling-death^b^	1.0 (0.3–4.4)	1.0 (0.4–3.5)	0.9 (0.2–5.4)	1.0 (0.3–4.4)	0.9 (0.2–8.5)
Thal phase					
0	79 (31.6)	75 (32.6)	19 (26.8)	24 (23.8)	14 (23.3)
1–2	73 (29.2)	70 (30.4)	18 (25.3)	27 (26.7)	14 (23.3)
3	47 (18.8)	45 (19.6)	16 (22.5)	19 (18.8)	11 (18.3)
4–5	51 (20.4)	40 (17.4)	18 (25.3)	31 (30.7)	21 (35.0)
Braak stage					
0	122 (48.8)	118 (51.3)	28 (39.4)	36 (35.6)	19 (31.7)
1–2	84 (33.6)	78 (33.9)	25 (35.2)	35 (34.6)	20 (33.3)
3–4	34 (13.6)	28 (12.2)	13 (18.3)	22 (21.8)	13 (21.7)
5–6	10 (4.0)	6 (2.6)	5 (7.0)	8 (7.9)	8 (13.3)
ADNC level					
Not AD	79 (31.6)	75 (32.6)	19 (26.8)	24 (23.8)	14 (23.3)
Low	129 (51.6)	122 (53.0)	34 (47.9)	49 (48.5)	26 (43.3)
Intermediate	33 (13.2)	28 (12.2)	13 (18.3)	20 (19.8)	12 (20.0)
High	9 (3.6)	5 (2.2)	5 (7.0)	8 (7.9)	8 (13.3)

a: expressed in years; b: expressed in months.

Continuous variables are reported as median (interquartile range). Categorical variables are reported as number (%). Abbreviations: AD, Alzheimer’s disease; ADNC, Alzheimer’s disease neuropathologic change; CSF, cerebrospinal fluid

Essential neuropathological features of included participants across subgroups are reported in Table 1. Across all subgroups, most subjects had either no ADNC (CSF cohort: 75/230, 32.6%; non-prion CSF cohort: 19/71, 26.8%; plasma cohort: 24/101, 23.8%; non-prion plasma cohort: 14/60, 23.3%) or low ADNC (CSF cohort: 122/230, 53.0%; non-prion CSF cohort: 34/71, 47.9%; plasma cohort: 49/101, 48.5%; non-prion plasma cohort: 26/60, 43.3%). Supplementary Table 1 presents the demographics of the non-prion subgroup with complete CSF biomarker availability (including CSF p-tau_217_, *n=*61). The primary neuropathological diagnoses of the included participants are detailed in Supplementary Table 2.

### Changes of CSF biomarkers in relation to Aβ and tau pathology burden

**Fig. 1:**
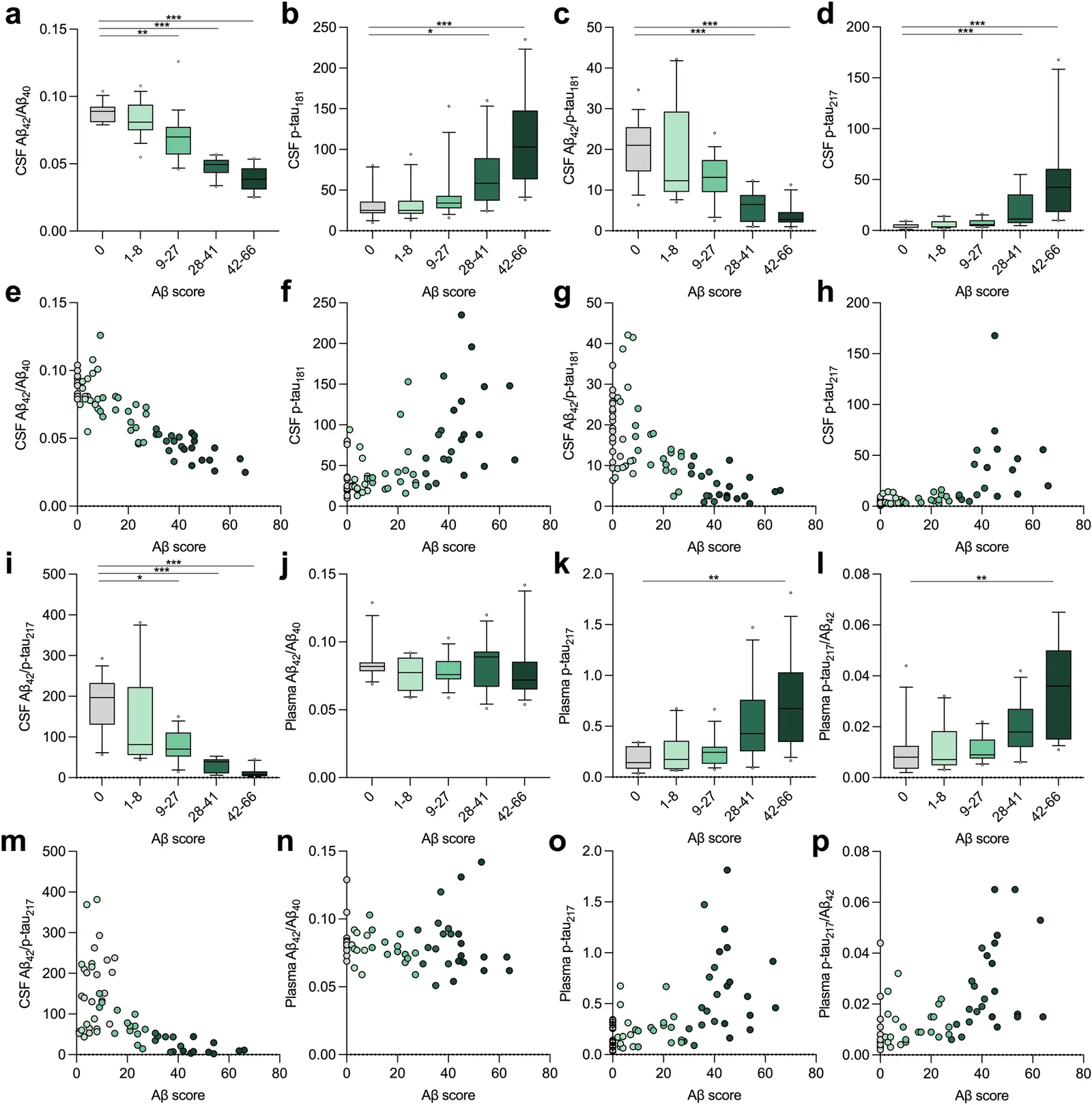
Distribution of biofluid biomarkers across increasing Aβ pathology burden in the non-prion subgroups. **a–d, i–l**: Box plots illustrating the distribution of CSF Aβ_42_/Aβ_40_ (**a**), p-tau_181_ (**b**), Aβ_42_/p-tau_181_ (**c**), p-tau_217_ (**d**), Aβ_42_/p-tau_217_ (**i**), plasma Aβ_42_/Aβ_40_ (**j**), p-tau_217_ (**k**) and p-tau_217_/Aβ_42_ (**l**) across quartiles of Aβ pathology score in the non-prion subgroups. The reference group (A*β =* 0) is shown in grey. Asterisks indicate significant differences compared with the reference group, based on multivariable linear regression models (adjusted for age at CSF/plasma sampling, sex and time between CSF/plasma sampling and death). Boxes represent the interquartile range, horizontal lines the median, whiskers the 10th–90th percentile range, and white circles outliers. **e**–**h**, **m–p**: Scatter plots showing the association between continuous values of Aβ score (x-axis) and levels of biofluid biomarkers. Color gradients indicate increasing Aβ pathology burden. For visualization, raw biomarker values were used, although log-transformed values were employed in the models. Abbreviations: CSF, cerebrospinal fluid

**Fig. 2:**
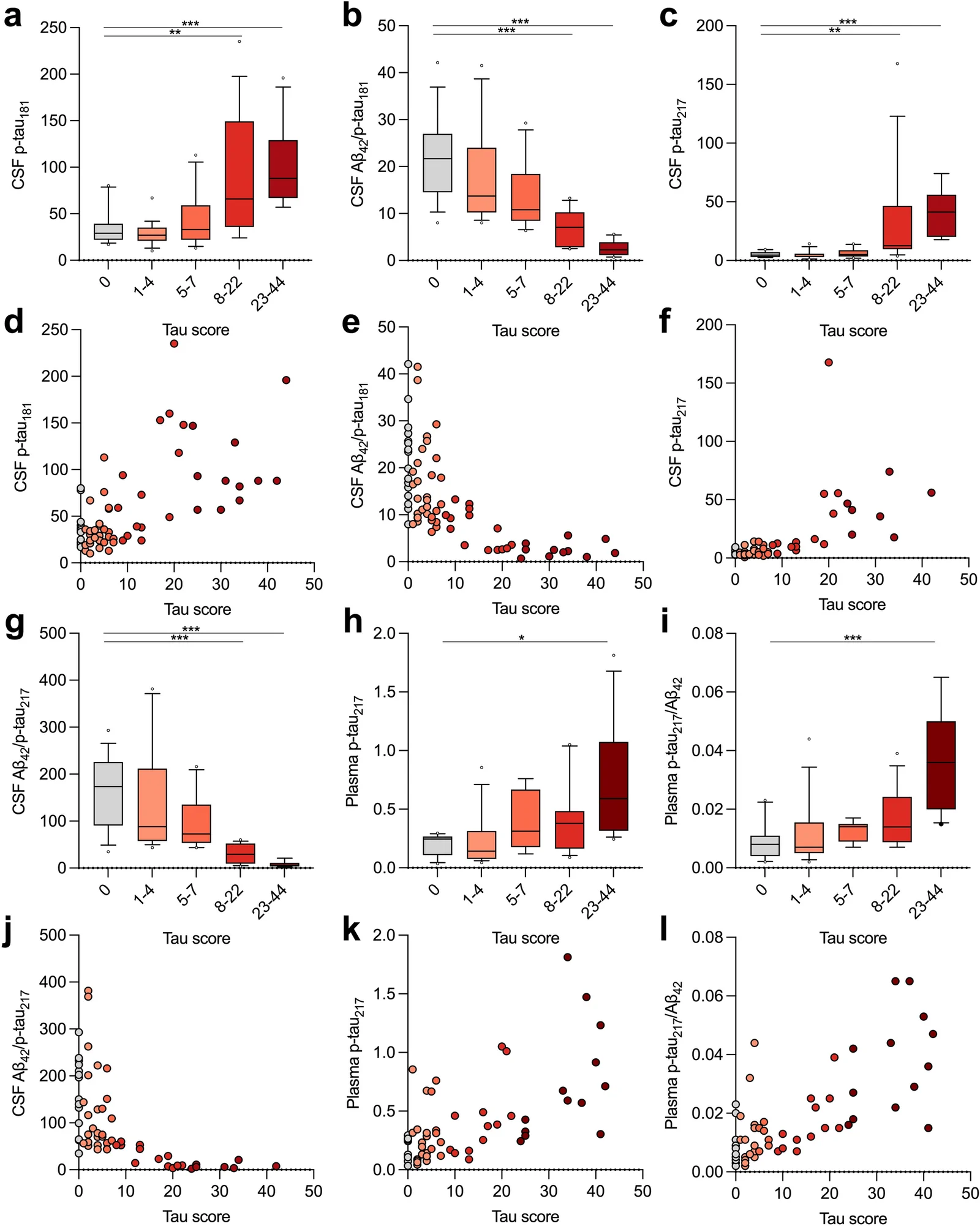
Distribution of biofluid biomarkers across increasing tau pathology burden in the non-prion subgroups. **a–c, g–i**: Box plots illustrating the distribution of CSF p-tau_181_ (**a**), Aβ_42_/p-tau_181_ (**b**), p-tau_217_ (**c**), Aβ_42_/p-tau_217_ (**g**), plasma p-tau_217_ (**h**) and p-tau_217_/Aβ_42_ (**i**) across quartiles of tau pathology score in the non-prion subgroups. The reference group (tau score = 0) is shown in grey. Asterisks indicate significant differences compared with the reference group, based on multivariable linear regression models (adjusted for age at CSF/plasma sampling, sex and time between CSF/plasma sampling and death). Boxes represent the interquartile range, horizontal lines the median, whiskers the 10th–90th percentile range, and white circles outliers. **d**-**f**, **j**-**l**: Scatter plots showing the association between continuous values of tau score (x-axis) and levels of biofluid biomarkers. Color gradients indicate increasing tau pathology burden. For visualization, raw biomarker values were used, although log-transformed values were employed in the models

The distribution of CSF biomarker values across quartiles of Aβ and tau pathology burden is shown in Table 2 and Fig. 1 (Aβ) and Fig. 2 (tau).

**Table 2:** Distribution of CSF biomarkers across quartiles of Aβ and tau pathology scores

CSF cohort (*n=*230)					
*Aβ score*					
	Reference (*n=*70)	1st quartile (*n=*42)	2nd quartile (*n=*48)	3rd quartile (*n=*32)	4th quartile (*n=*38)
*Aβ score* Range Median (IQR)	0–0 0 (0–0)	1–7 4 (2–5)	8–21 14 (10–17.7)	22–35 27 (24–31)	36–66 43.5 (38–48.2)
Aβ_42_/Aβ_40_	0.090 (0.082–0.095)	0.084 (0.078–0.092)	0.079 (0.071–0.086)	0.057 (0.047–0.068)	0.048 (0.036–0.054)

Non-prion CSF cohort (*n=*71)					
*Aβ score*					
	Reference (*n=*17)	1st quartile (*n=*15)	2nd quartile (*n=*17)	3rd quartile (*n=*10)	4th quartile (*n=*12)
*Aβ score* Range Median (IQR)	0–0 0 (0–0)	1–8 4 (3–7)	9–27 21 (12.5–24)	28–41 36.5 (31.7–38.5)	42–66 47.5 (45–54)
Aβ_42_/Aβ_40_	0.089 (0.081–0.092)	0.081 (0.075–0.094)	0.070 (0.057–0.077)	0.049 (0.043–0.053)	0.038 (0.031–0.047)
p-tau_181_	25.0 (21.5–36.0)	25.0 (21.0–37.0)	34.0 (27.5–43.0)	58.5 (37.0–89.2)	103.0 (63.2–147.8)
Aβ_42_/p-tau_181_	21.0 (14.6–25.5)	12.3 (9.5–29.3)	13.1 (9.5–17.4)	6.5 (2.2–8.8)	2.7 (2.1–4.6)
p-tau_217_	3.0 (2.6–6.1)	3.5 (2.9–9.1)	5.7 (4.3–10.0)	11.2 (7.2–35.3)	42.4 (18.0–60.6)
Aβ_42_/p-tau_217_	196.7 (130.2–232.6)	81.4 (55.5–222.7)	70.3 (52.0–111.2)	39.0 (10.8–45.2)	8.3 (3.6–15.6)

*Tau score*					
	Reference (*n=*16)	1st quartile (*n=*19)	2nd quartile (*n=*11)	3rd quartile (*n=*14)	4th quartile (*n=*11)
*Tau score* Range Median (IQR)	0–0 0 (0–0)	1–4 2 (2–4)	5–7 6 (5–6)	8–22 13 (9.7–19.2)	23–44 33 (25–38)
p-tau_181_	29.0 (22.0–39.2)	27.0 (21.0–35.0)	33.0 (22.0–59.0)	66.0 (35.7–149.3)	88.0 (67.0–129.0)
Aβ_42_/p-tau_181_	21.6 (14.5–26.9)	13.7 (10.2–24.0)	10.8 (8.4–19.4)	7.0 (2.8–10.3)	2.3 (1.1–3.9)
p-tau_217_	4.5 (2.9–7.1)	3.4 (2.8–5.7)	5.1 (3.4–8.8)	12.6 (9.6–46.6)	41.2 (20.1–56.1)
Aβ_42_/p-tau_217_	173.3 (90.8–226.0)	88.2 (57.7–211.9)	72.6 (53.5–135.4)	29.2 (9.2–52.5)	6.2 (3.5–11.1)

Biomarker values are reported as median (interquartile range). Values of p-tau_181_ and p-tau_217_ are expressed in pg/ml. Values of p-tau_217_ and Aβ_42_/p-tau_217_ were available in a subgroup (*n=*61). Abbreviations: CSF, cerebrospinal fluid; IQR, interquartile range

In models that included Aβ or tau pathology score as the sole predictor, all five CSF biomarkers were associated with both Aβ and tau pathology scores (all *p<*0.001). However, when both Aβ and tau scores were included in the same model, CSF Aβ_42_/Aβ_40_ showed a stronger association with Aβ pathology (*β =* − 0.01, 95%CI − 0.01 to − 0.01, *p<*0.001) than with tau pathology (*β =* − 0.004, 95%CI − 0.01 to − 0.001, *p=*0.021). Similar results were obtained in the non-prion CSF cohort, where the association remained significant only for Aβ pathology (*β =* − 0.01, 95%CI − 0.02 to − 0.01, *p<*0.001). Conversely, CSF p-tau_181_ was independently associated only with tau pathology (*β =* 0.03, 95%CI 0.01 to 0.05, *p=*0.002), whereas CSF p-tau_217_, Aβ_42_/p-tau_181_ and Aβ_42_/p-tau_217_ were independently related to continuous scores of both pathologies (Supplementary Table 3).

Increasing Aβ pathology burden was associated with progressive changes in CSF biomarkers. Specifically, CSF Aβ_42_/Aβ_40_ showed the earliest significant decrease at the second quartile of Aβ pathology (*β =* − 0.11, 95%CI − 0.19 to − 0.04, *p=*0.001), with progressively greater reductions in the third (*β =* − 0.42, 95%CI − 0.51 to − 0.34) and fourth quartiles (*β =* − 0.62, 95%CI − 0.71 to − 0.54, both *p<*0.001). These findings were replicated in the non-prion CSF cohort (second quartile: *β =* − 0.23, 95%CI − 0.36 to − 0.09, *p=*0.001; third quartile: *β =* − 0.57, 95%CI − 0.73 to − 0.41, *p<*0.001; fourth quartile: *β =* − 0.80, 95%CI − 0.95 to − 0.65, *p<*0.001) (Fig. 1). Similarly, CSF Aβ_42_/p-tau_217_ levels displayed the first significant decrease at the second quartile of Aβ pathology (*β =* − 0.65, 95%CI − 1.20 to − 0.11, *p=*0.020). Conversely, a significant increase in CSF p-tau_181_ and p-tau_217_, and a reduction in Aβ_42_/p-tau_181_, emerged only at higher Aβ pathology burdens, specifically in the third quartile (p-tau_181_: *β =* 0.64, 95%CI 0.15 to 1.13, *p=*0.011; p-tau_217_: *β =* 1.19, 95%CI 0.52 to 1.86, *p<*0.001; Aβ_42_/p-tau_181_: *β =* − 1.33, 95%CI − 1.86 to − 0.79, *p<*0.001). The magnitude of changes of these markers increased further in the highest quartile of Aβ pathology score (Fig. 1, Supplementary Table 4). All results were replicated in the subgroup of participants with complete CSF biomarker availability, except for Aβ_42_/p-tau_181_, which was already significantly reduced in the second quartile (Supplementary Table 5).

In relation to tau pathology burden, the earliest significant increases in CSF p-tau_181_ and p-tau_217_ and reductions in Aβ_42_/p-tau_181_ and Aβ_42_/p-tau_217,_ emerged in the third quartile (p-tau_181_: *β =* 0.82, 95%CI 0.32 to 1.32, *p=*0.001; p-tau_217_: *β =* 1.12, 95%CI 0.41 to 1.83, *p=*0.002; Aβ_42_/p-tau_181_: *β =* − 1.16, 95%CI − 1.64 to − 0.67, *p<*0.001; Aβ_42_/p-tau_217_: *β =* − 1.44, 95%CI − 2.14 to − 0.74, *p<*0.001). Greater tau pathology burden was associated with larger biomarker changes (Fig. 2, Supplementary Table 4). These findings were replicated after exclusion of participants with PART (Supplementary Table 6). Moreover, changes in p-tau_181_, Aβ_42_/p-tau_181_ and Aβ_42_/p-tau_217_ in relation to tau pathology levels remained consistent after adjusting for the Aβ score (Supplementary Table 7).

The changes in CSF biomarkers in relation to standard neuropathological staging systems are reported in Supplementary Tables 8–10. Specifically, reductions in CSF Aβ_42_/Aβ_40_ were already evident at low ADNC level, whereas significant changes in CSF p-tau_181_, p-tau_217_, Aβ_42_/p-tau_181_ and Aβ_42_/p-tau_217_ started at intermediate ADNC level (Supplementary Table 10).

### Discriminatory performance of CSF biomarkers across increasing Aβ and tau pathology burden

**Fig. 3:**
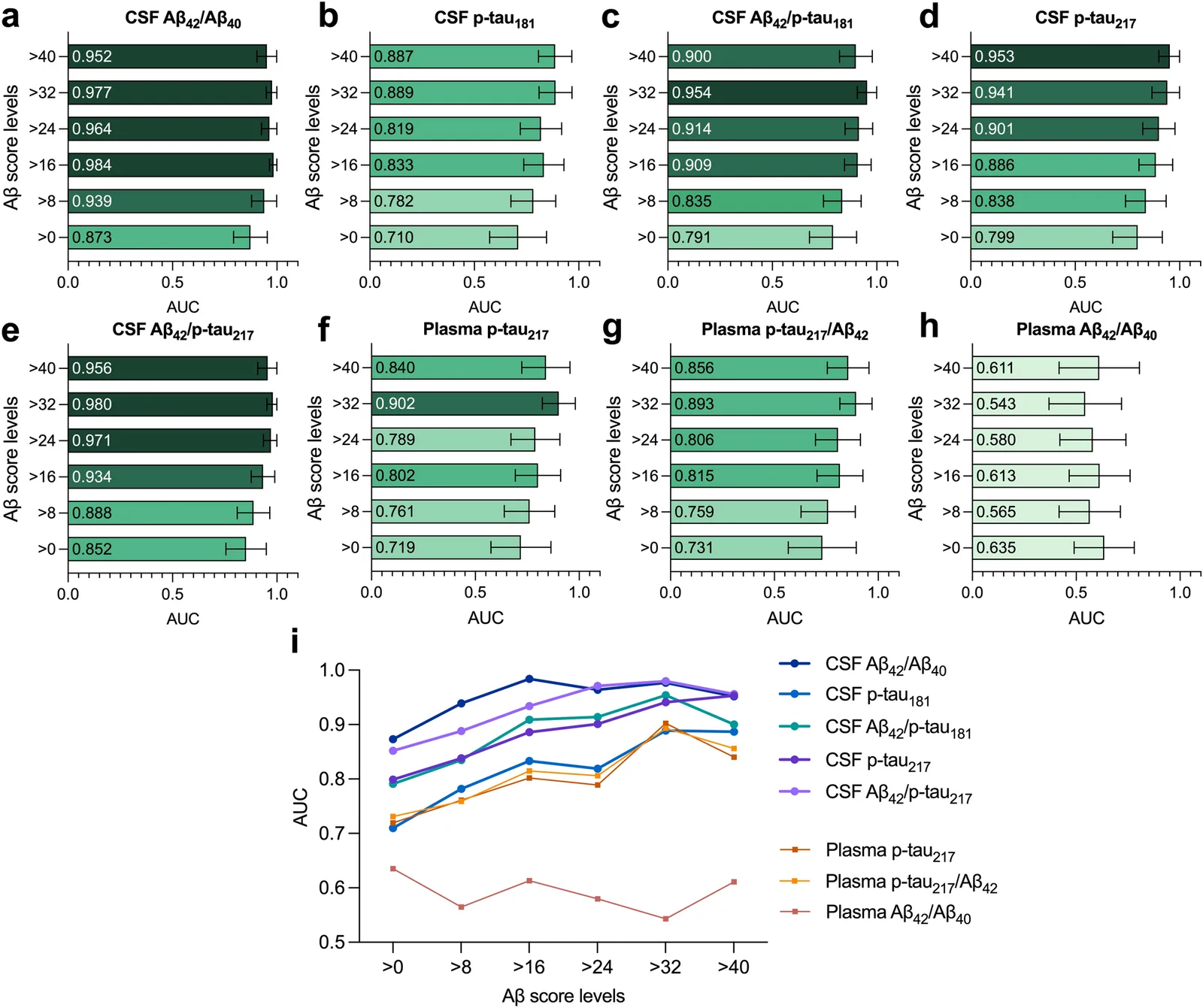
Discriminatory performance of biofluid biomarkers across increasing Aβ pathology burden in the non-prion subgroups. **a–h:** Bar plots of AUC values for each biomarker for discriminating progressively higher Aβ burden in the non-prion subgroups, defined by increasing Aβ score thresholds (y-axis). Error bars represent 95% confidence intervals. Increasing shades of green reflect higher AUC values. **i**: Line plot showing AUC values across Aβ score thresholds for all biomarkers. 95% confidence intervals are not shown for graphical reasons. Abbreviations: AUC, area under the curve

Next, we performed sequential ROC analyses across increasing levels of Aβ pathology and tau burden to evaluate the discriminatory performance of CSF biomarkers at different stages of AD pathology. The accuracy of CSF Aβ_42_/Aβ_40_ progressively increased with Aβ score levels, reaching the highest AUC values at intermediate Aβ score thresholds (> 24 in the entire CSF cohort, area under the curve (AUC) 0.966, 95%CI 0.943 to 0.989; >16 in the non-prion subgroup, AUC 0.984, 95%CI 0.964 to 1.000). In contrast, CSF p-tau_181_, p-tau_217_, Aβ_42_/p-tau_181_ and Aβ_42_/p-tau_217_ achieved the highest discriminatory performance at higher thresholds of Aβ pathology burden. Specifically, p-tau_181_ and p-tau_217_ reached their highest AUC values at Aβ scores >32 (p-tau_181_: AUC 0.889, 95%CI 0.810 to 0.968; p-tau_217_: AUC 0.941, 95%CI 0.868 to 1.000) and >40 (p-tau_181_: AUC 0.887, 95%CI 0.807 to 0.967; p-tau_217_: AUC 0.953, 95%CI 0.901 to 1.000), while Aβ_42_/p-tau_181_ and Aβ_42_/p-tau_217_ showed the highest performance at Aβ scores >32 (Aβ_42_/p-tau_181_: AUC 0.954, 95%CI 0.907 to 1.000; Aβ_42_/p-tau_217_: AUC 0.980, 95%CI 0.952 to 1.000) (Fig. 3). Overall, CSF p-tau_181_ demonstrated lower discrimination power than CSF Aβ_42_/Aβ_40_ across the Aβ pathology continuum. CSF Aβ_42_/p-tau_181_ and p-tau_217_ performed slightly better, although their accuracy remained generally inferior to Aβ_42_/Aβ_40_ throughout the entire Aβ pathology continuum. Interestingly, CSF Aβ_42_/p-tau_217_ yielded AUC values comparable to those of Aβ_42_/Aβ_40_ at most Aβ score thresholds (Fig. 3, Supplementary Table 11).

**Fig. 4:**
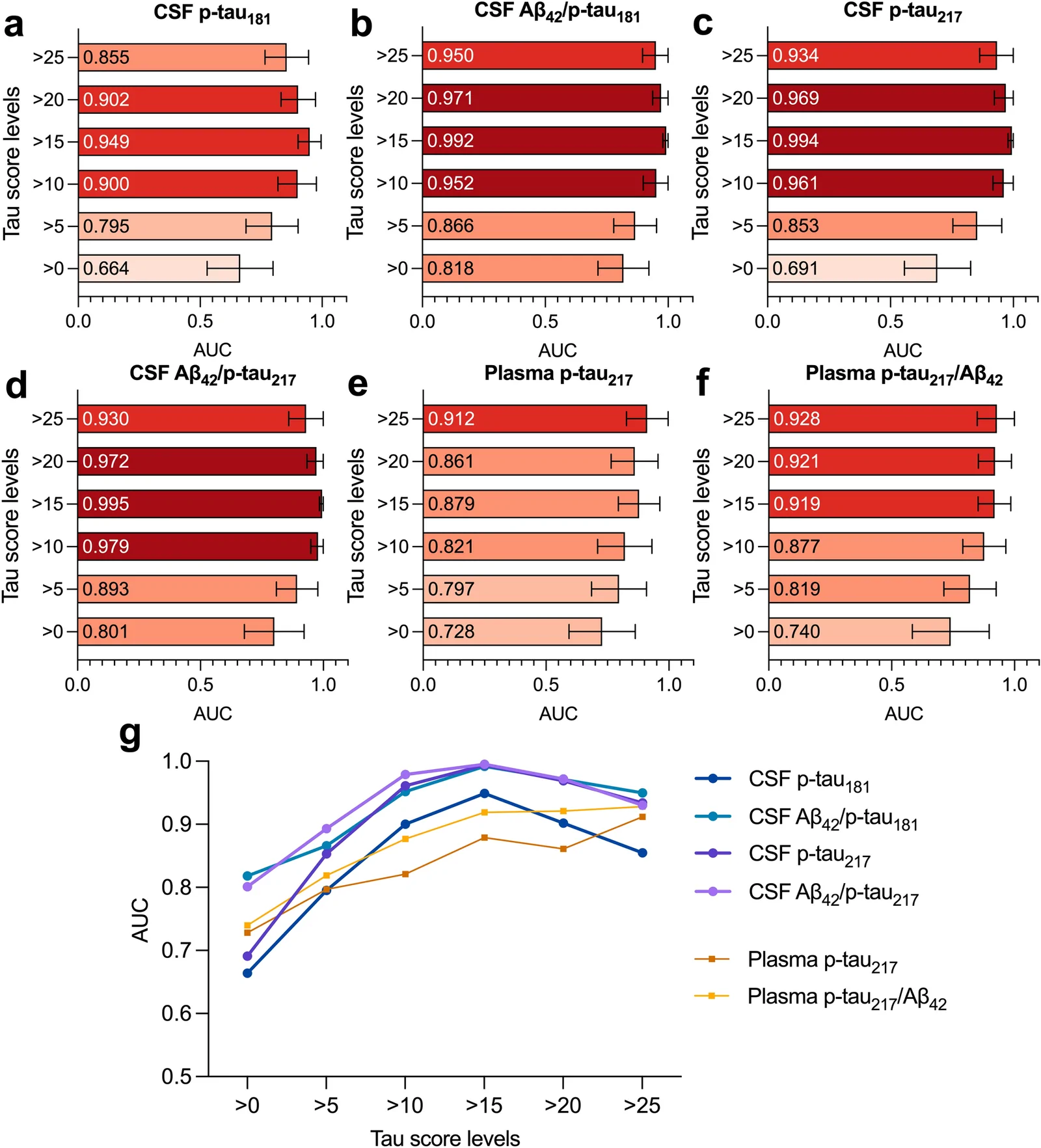
Discriminatory performance of biofluid biomarkers across increasing tau pathology burden in the non-prion subgroups. **a**–**f**: Bar plots of AUC values for each biomarker for discriminating progressively higher tau burden in the non-prion subgroups, defined by increasing tau score thresholds (y-axis). Error bars represent 95% confidence intervals. Increasing shades of red reflect higher AUC values. **g:** Line plot showing AUC values across tau score thresholds for all biomarkers. 95% confidence intervals are not shown for graphical reasons. Abbreviations: AUC, area under the curve

Regarding the tau pathology load, CSF p-tau_181_, p-tau_217_, Aβ_42_/p-tau_181_ and Aβ_42_/p-tau_217_ yielded the highest discrimination power at intermediate levels of pathology burden, specifically at a tau score >15 (p-tau_181_: AUC 0.949, 95%CI 0.902 to 0.996; p-tau_217_: AUC 0.994, 95%CI 0.979 to 1.000; Aβ_42_/p-tau_181_: AUC 0.992, 95%CI 0.979 to 1.000; Aβ_42_/p-tau_217_: AUC 0.995, 95%CI 0.984 to 1.000) (Fig. 4, Supplementary Table 12). The overall discriminatory performance patterns observed for CSF biomarkers across increasing Aβ and tau pathology burdens were largely replicated in the subgroup of participants with complete biomarker data available (Supplementary Figs. 1 and 2). Cut-off values of CSF biomarkers corresponding to 95% specificity for detecting Aβ and tau burden above the different thresholds are reported in Supplementary Tables 11 and 12.

The performance of CSF biomarkers across categories of standard neuropathological staging systems, including ADNC, is reported in Supplementary Tables 13–15. CSF Aβ_42_/Aβ_40_, p-tau_217_, Aβ_42_/p-tau_181_ and Aβ_42_/p-tau_217_ demonstrated excellent performance (all AUCs >0.960) in discriminating participants with intermediate/high ADNC. Notably, in the non-prion CSF cohort, a cut-off <0.074 achieved 95% specificity for distinguishing participants with at least a low ADNC level (Supplementary Table 15).

### Changes of plasma biomarkers in relation to Aβ and tau pathology burden

The distribution of plasma biomarker values across quartiles of Aβ and tau pathology burden is shown in Table 3 and Fig. 1 (Aβ) and Fig. 2 (tau).

**Table 3:** Distribution of plasma biomarkers across quartiles of Aβ and tau pathology scores

Plasma cohort (*n=*101)					
*Aβ score*					
	Reference (*n=*23)	1st quartile (*n=*17)	2nd quartile (*n=*18)	3rd quartile (*n=*17)	4th quartile (*n=*26)
*Aβ score* Range Median (IQR)	0–0 0 (0–0)	1–7 4 (3–6)	8–21 15.5 (10.7–19.2)	22–35 28.0 (26.5–30.5)	36–64 45 (40–49.5)
Aβ_42_/Aβ_40_	0.081 (0.078–0.086)	0.081 (0.076–0.092)	0.079 (0.075–0.088)	0.073 (0.067–0.076)	0.080 (0.068–0.090)

Non-prion plasma cohort (*n=*60)					
*Aβ score*					
	Reference (*n=*13)	1st quartile (*n=*10)	2nd quartile (*n=*13)	3rd quartile (*n=*11)	4th quartile (*n=*13)
*Aβ score* Range Median (IQR)	0–0 0 (0–0)	1–8 3.5 (3–6.2)	9–27 20 (12.5–23.5)	28–41 36 (32–40)	42–64 46 (44.5–54)
Aβ_42_/Aβ_40_	0.082 (0.078–0.085)	0.077 (0.064–0.088)	0.076 (0.072–0.086)	0.089 (0.067–0.093)	0.072 (0.065–0.085)
p-tau_217_	0.143 (0.082–0.304)	0.174 (0.077–0.357)	0.244 (0.131–0.300)	0.427 (0.254–0.760)	0.674 (0.345–1.031)
p-tau_217_/Aβ_42_	0.008 (0.003–0.012)	0.007 (0.005–0.018)	0.009 (0.007–0.015)	0.018 (0.012–0.027)	0.036 (0.015–0.050)

*Tau score*					
	Reference (*n=*11)	1st quartile (*n=*17)	2nd quartile (*n=*7)	3rd quartile (*n=*12)	4th quartile (*n=*13)
*Tau score* Range Median (IQR)	0–0 0 (0–0)	1–4 3 (2–4)	5–7 6 (5–7)	8–22 16 (10.7–19.7)	23–42 34 (25–40.5)
p-tau_217_	0.244 (0.107–0.266)	0.143 (0.076–0.314)	0.313 (0.177–0.667)	0.379 (0.164–0.483)	0.591 (0.315–1.075)
p-tau_217_/Aβ_42_	0.008 (0.004–0.011)	0.007 (0.005–0.015)	0.014 (0.009–0.015)	0.014 (0.009–0.024)	0.036 (0.020–0.050)

Biomarker values are reported as median (interquartile range). Values of p-tau_217_ are expressed in pg/ml

*IQR* Interquartile range

In models that included continuous Aβ or tau pathology scores as the sole predictor, plasma p-tau_217_ and p-tau_217_/Aβ_42_ were associated with both Aβ and tau pathology scores (all *p<*0.001). However, when we included both scores as predictors in the same model, plasma p-tau_217_ and p-tau_217_/Aβ_42_ remained independently associated only with tau pathology (p-tau_217_: *β =* 0.02, 95%CI 0.004 to 0.04, *p=*0.019; p-tau_217_/Aβ_42_: *β =* 0.03, 95%CI 0.01 to 0.05, *p<*0.001). In contrast, plasma Aβ_42_/Aβ_40_ showed weak associations with both Aβ (*β =* − 0.003, 95%CI − 0.006 to − 0.0004, *p=*0.026) and tau (*β =* 0.005, 95%CI 0.001 to 0.01, *p=*0.047) pathology scores in the entire plasma cohort. Still, these associations were lost in the non-prion subgroup (Supplementary Table 3).

In relation to Aβ pathology burden, the first significant increase in plasma p-tau_217_ and p-tau_217_/Aβ_42_ only emerged in the highest quartile (p-tau_217_: *β =* 0.86, 95%CI 0.26 to 1.46, *p=*0.005; p-tau_217_/Aβ_42_: *β =* 0.91, 95%CI 0.32 to 1.51, *p=*0.003). In contrast, plasma Aβ_42_/Aβ_40_ showed no significant stepwise changes across increasing quartiles of Aβ pathology burden (Fig. 1, Supplementary Table 4).

Significant changes in plasma p-tau_217_ and p-tau_217_/Aβ_42_ levels were also observed at the highest quartile of tau pathology score (p-tau_217_: *β =* 0.68, 95%CI 0.06 to 1.29, *p=*0.032; p-tau_217_/Aβ_42_: *β =* 1.05, 95%CI 0.48 to 1.62, *p<*0.001) (Fig. 2, Supplementary Table 4). These findings were replicated after excluding the participants with PART (Supplementary Table 6).

Changes in plasma biomarkers across levels of standard neuropathological staging systems are reported in Supplementary Tables 8–10. These analyses confirmed a significant increase in plasma p-tau_217_ and p-tau_217_/Aβ_42_ at later stages of the AD continuum, particularly among participants with high ADNC (Supplementary Table 10).

### Discriminatory performance of plasma biomarkers across increasing Aβ and tau pathology burden

The discrimination power of p-tau_217_ and p-tau_217_/Aβ_42_ increased across higher levels of Aβ burden, reaching peak performance at elevated pathology loads (i.e., Aβ score >32) (p-tau_217_: AUC 0.902, 95%CI 0.823 to 0.981; p-tau_217_/Aβ_42_: AUC 0.893, 95%CI 0.815 to 0.970). In contrast, plasma Aβ_42_/Aβ_40_ showed poor discriminatory performance across the entire Aβ pathology continuum (Fig. 3, Supplementary Table 11).

Similarly, the discriminatory performance of plasma p-tau_217_ and p-tau_217_/Aβ_42_ increased across progressively higher tau pathology thresholds, with the highest AUC values observed at tau score >25 (p-tau_217_: AUC 0.912, 95%CI 0.827 to 0.997; p-tau_217_/Aβ_42_: AUC 0.928, 95%CI 0.848 to 1.000) (Fig. 4, Supplementary Table 12). The overall discriminatory performance patterns observed for plasma biomarkers across increasing Aβ and tau pathology burdens were largely replicated in the subgroup of participants with complete biomarker data available (Supplementary Figs. 1 and 2). The cut-off values of plasma biomarkers corresponding to 95% specificity for detecting Aβ and tau burden above the different thresholds are reported in Supplementary Tables 11 and 12.

The performance of plasma biomarkers across categories of standard neuropathological staging systems, including ADNC, is reported in Supplementary Tables 13–15. Plasma p-tau_217_/Aβ_42_ slightly outperformed p-tau_217_ alone in discriminating participants with intermediate/high ADNC (AUCs 0.879 vs 0.831) (Supplementary Table 15).

## Discussion

In this study, we comprehensively characterize the relationship between Aβ and tau neuropathological burden and corresponding changes in fluid biomarkers across the AD continuum, leveraging a cohort with CSF and plasma samples taken shortly before death.

CSF Aβ_42_/Aβ_40_ ratio showed the earliest changes, with significantly reduced levels already detectable in subjects with low-intermediate Aβ pathology, typically corresponding to mild-to-moderate Aβ deposition across neocortical and limbic regions. Increasing Aβ burden was associated with progressively greater reductions in CSF Aβ_42_/Aβ_40_. Consistent with its role as an early marker, CSF Aβ_42_/Aβ_40_ demonstrated the highest discrimination accuracy at low-to-intermediate levels of Aβ pathology (i.e., Aβ score >24 in the entire CSF cohort and >16 in the non-prion subgroup), followed by a modest decrease at higher pathological thresholds, suggesting a plateau effect at more advanced stages. Despite the high sensitivity for detecting Aβ pathology, CSF Aβ_42_/Aβ_40_ showed limited discrimination accuracy at the very earliest stages of plaque formation (i.e., mild neocortical deposits), suggesting the presence of an initial window in the AD continuum that current CSF biomarkers may not fully capture. These findings are consistent with previous neuropathological studies reporting reductions in CSF Aβ_42_/Aβ_40_ at Thal phases 2–3 corresponding approximately to the second quartile of Aβ pathology score in our cohort [8, 34], and with evidence indicating that CSF Aβ_42_/Aβ_40_ typically becomes abnormal before detectable increases in amyloid PET signal, which generally occur at more advanced Thal phases [17, 43, 52].

Despite its wide use in clinical practice, neuropathological validation studies of the CSF Aβ_42_/Aβ_40_ ratio are scarce [8, 34]. Here, we showed that a CSF Aβ_42_/Aβ_40_ cut-off of <0.074, slightly higher than that commonly used in clinical settings, provides 95% specificity in identifying subjects with ongoing Aβ pathology (i.e., at least low ADNC) regardless of its severity, thereby supporting its potential utility for the early identification of subjects within the AD continuum. Notably, the similar associations between CSF Aβ_42_/Aβ_40_ and Aβ burden in the entire CSF cohort and in the non-prion subgroup suggest that CSF Aβ_42_/Aβ_40_ reliably reflects Aβ pathology even in the presence of a significant co-pathology.

Regarding the “p-tau” markers, our results suggest that CSF p-tau_181_ is more closely associated with later stages of the AD continuum, given that a significant increase in its levels is only detected in association with an intermediate-to-high Aβ burden. Furthermore, changes in CSF p-tau_181_ became evident only in the presence of concomitant moderate tau pathology, even after accounting for Aβ pathology load and excluding participants with PART. Consistently, CSF p-tau_181_ was independently associated with continuous tau pathology scores, not with Aβ scores, when both pathologies were included in the same model. In contrast, CSF p-tau_217_ retained an independent association with Aβ pathology, consistent with previous Aβ- and tau-PET studies demonstrating a stronger association with Aβ pathology for CSF p-tau_217_ than for CSF p-tau_181_ [21, 30]. Although both CSF p-tau_217_ and p-tau_181_ showed a significant increase at a comparable level of Aβ burden, p-tau_217_ levels demonstrated an elevation trend already at the second quartile of Aβ pathology, consistent with previous evidence suggesting that tau phosphorylation at threonine 217 may occur earlier than phosphorylation at threonine 181 during AD pathogenesis [21, 32]. Altogether, these results align with previous neuropathological observations indicating that increases in CSF p-tau biomarkers occur downstream of the earliest alterations in CSF Aβ_42_/Aβ_40_ [15, 28, 34, 45, 59].

When considering the combined ratios, reductions in Aβ_42_/p-tau_181_ and Aβ_42_/p-tau_217_ were already detectable at low-to-intermediate Aβ burden (i.e., second quartile), similar to Aβ_42_/Aβ_40_. This was evident in the primary analysis for Aβ_42_/p-tau_217_ and in the sensitivity analysis restricted to participants with complete CSF biomarker availability for Aβ_42_/p-tau_181_ calculation. However, abnormalities in both CSF Aβ_42_/p-tau ratios, as well as in CSF p-tau_181_ and p-tau_217_, emerged only from intermediate ADNC levels, unlike CSF Aβ_42_/Aβ_40,_ which was already reduced at low ADNC. These observations support the notion that CSF Aβ_42_/p-tau hybrid ratios reflect more advanced AD pathology than CSF Aβ_42_/Aβ_40_ ratio, likely because they integrate two distinct pathogenic mechanisms.

Consistently, we showed that the discriminatory performance of CSF Aβ_42_/p-tau_181_ and Aβ_42_/p-tau_217_ ratios in detecting different levels of Aβ and tau burden progressively increased across increasing thresholds, peaking at high Aβ and intermediate tau scores. Notably, CSF Aβ_42_/p-tau_217_ outperformed Aβ_42_/p-tau_181_ at most Aβ pathology thresholds, particularly at intermediate-high burden levels, where its performance approached that of Aβ_42_/Aβ_40_. Similarly, discrimination of increasing tau burden was generally higher for CSF Aβ_42_/p-tau_217_ than for Aβ_42_/p-tau_181_. Altogether, these findings suggest that CSF Aβ_42_/p-tau_217_ may be a more accurate integrated marker of combined Aβ and tau pathology than Aβ_42_/p-tau_181_. Moreover, Aβ_42_/p-tau hybrid ratios generally performed better than the corresponding p-tau markers alone for both Aβ and tau thresholds, further supporting the use of biomarker ratios over single-marker measurements in clinical settings.

In plasma, our results suggest these markers reflect later stages of the AD continuum than CSF markers. Specifically, independent increases in plasma p-tau_217_ and p-tau_217_/Aβ_42_ were detectable only at an advanced Aβ and tau pathology burden, corresponding to the highest pathology quartiles and high ADNC. Moreover, the discriminatory performance of both markers progressively increased with higher Aβ and tau pathology burden, reaching peak accuracy at the highest pathological thresholds.

Previous neuropathological studies have yielded inconsistent findings regarding the relationship between plasma p-tau_217_ levels and the stage of AD pathology. While some studies indicated that plasma p-tau_217_ may already be elevated at intermediate Braak stages [9, 27], other studies reported significant abnormalities only at advanced stages of conventional AD neuropathological staging systems [54, 56, 58]. Similarly, a recent tau-PET-based study showed that plasma p-tau_217_ levels, measured through the Lumipulse platform, became abnormal from estimated Braak stages III-IV onward, while showing the highest discriminatory value for identifying subjects with neocortical tau pathology (i.e., Braak stages ≥4) [14]. These findings are consistent with our results and support the notion that substantial Aβ and tau pathological cortical burden may be required before significant group-level independent elevations in plasma p-tau_217_ become detectable. In line with this interpretation, a marked increase in soluble p-tau_217_ release has been linked to the spread of tau pathology into the neocortex [33]. Consistently, plasma p-tau_217_ has been shown to be strongly associated with measures of tau pathology burden, including both neuropathological load and PET signal, in subjects at advanced disease stages [33]. This may explain why plasma p-tau_217_ in our cohort was independently associated only with tau pathology when we modelled both proteinopathies simultaneously. Additionally, approximately one-third of our plasma cohort had at least intermediate ADNC, unlike the CSF cohort, and this should be considered when interpreting the results.

Growing evidence indicates that, compared with CSF markers, plasma biomarker measurements are more susceptible to peripheral influences, particularly impaired kidney function and systemic comorbidities [24, 36, 41]. In addition, cross-reactivity of analytical antibodies with peripherally derived tau species may further affect plasma biomarker measurements [10, 26, 55]. Notably, accumulating evidence suggests that using ratios (e.g., p-tau_217_/Aβ_42_) rather than single biomarkers may reduce the influence of peripheral confounders on plasma biomarker performance [3, 29, 41]. However, the similar associations we observed in our study between plasma p-tau_217_ and p-tau_217_/Aβ_42_, and both Aβ and tau pathology burden levels seem to exclude a major influence of systemic confounders.

When we evaluated the plasma marker performance against conventional neuropathological staging systems, plasma p-tau_217_ showed good accuracy in discriminating subjects with intermediate/high ADNC (AUC 0.831), with further improvement in distinguishing subjects with high ADNC. Although this performance lies within the lower range of AUC values reported in previous neuropathological studies [27, 39, 40, 46, 54, 56, 58, 61], differences in cohort composition may partially explain this discrepancy. In particular, several of the cited studies were enriched for subjects with high ADNC compared with our cohort, potentially favoring higher discriminatory performance.

The limited neuropathological associations and poor discriminatory performance of plasma Aβ_42_/Aβ_40_ observed in our cohort warrant further discussion. Previous studies have reported a moderate performance of plasma Aβ_42_/Aβ_40_ in identifying Aβ+ participants defined by CSF or PET positivity when assessed through mass spectrometry-based methods [13, 20, 23, 47]. Some previous neuropathological studies have shown significant associations with semi-quantitative measures of Aβ burden or conventional AD neuropathological stages [11, 46]. However, other studies also reported weak or absent associations between plasma Aβ_42_/Aβ_40_ and post-mortem assessed AD pathology [50, 59]. Notably, none of the cited studies used the same analytical platform as our study. In our cohort, plasma Aβ_42_/Aβ_40_ showed only weak associations with both Aβ and tau pathology burden, together with limited discriminatory performance across increasing pathology thresholds. Several hypotheses can be raised, including greater influence of systemic confounders on Aβ_42_ and Aβ_40_ than on p-tau_217_, peripheral contributions to circulating Aβ_42_ and Aβ_40_ levels, and potential effects of blood–brain barrier integrity on plasma biomarker dynamics. Future studies in independent neuropathological cohorts will be needed to assess further the performance of Lumipulse plasma Aβ_42_/Aβ_40_ across different levels of AD pathological burden.

The major strengths of our study are the large neuropathological cohort, the detailed semi-quantitative assessment of Aβ pathology (including both parenchymal and vascular deposition) and tau pathology, and the short median interval between biofluid sampling and death. Moreover, the comprehensive characterization of neuropathological correlates of five CSF and three plasma biomarkers, including both single analytes and biomarker ratios, is a significant and original contribution to the literature on neuropathological biomarker validation. Finally, the cohort’s enrichment with a high proportion of subjects with low ADNC allowed us to investigate biomarker alterations during early AD stages, which remain underrepresented in most neuropathological studies.

Nevertheless, several limitations remain. First, the subgroup of participants without prion disease was relatively small, although still substantial considering the neuropathological nature of the study. In addition, the cohort largely comprised subjects with rapidly progressive neurological syndromes, which may limit the generalizability of our findings to broader clinical populations, particularly for plasma biomarkers. In this regard, we cannot exclude that the severe disability occurring in some of the patients at the time of sampling may have been partially influenced by systemic comorbidities. Unfortunately, kidney function data were unavailable for most participants, which is a limitation of our study. Moreover, although stratifying pathology scores into quartiles ensured comparisons across numerically balanced groups, using predefined quartile cut-offs prevented us from determining precise pathological burden thresholds associated with biomarker changes. Finally, we measured all biomarkers using a single analytical platform. While this ensured analytical homogeneity across measurements, it may limit the generalizability of our findings to other assay platforms and precludes direct inter-platform comparisons for the same analytes.

Altogether, our findings support a sequential model of biofluid biomarker abnormalities across the AD pathological continuum. CSF Aβ_42_/Aβ_40_ became abnormal in the early stages of Aβ deposition and best distinguished subjects at low-to-intermediate Aβ burden. CSF p-tau_181_ and p-tau_217_, as well as their hybrid ratios with Aβ_42_, reflected more advanced AD stages and performed best at high Aβ and intermediate tau burden. Plasma p-tau_217_ and p-tau_217_/Aβ_42_ became abnormal only in subjects with high Aβ and tau burden. At the same time, our results also suggest that the earliest phases of Aβ deposition may still not be fully captured by currently available biofluid markers.

## Supplementary Information

Below is the link to the electronic supplementary material.

Supplementary file1 (PDF 1060 KB)

## Data Availability

The dataset used and analyzed during the current study is available from the corresponding author upon reasonable request.
