# Supplementary material for: Changes in five cerebrospinal fluid and three plasma Alzheimer’s disease biomarkers across increasing brain amyloid-beta and tau pathology burden
Source: Acta Neuropathol. 2026 Sep 3;152(1):28. doi: 10.1007/s00401-026-03076-5 (PMC13541809; doi:10.1007/s00401-026-03076-5)
Supplement: Supplementary file 1 [file 401_2026_3076_MOESM1_ESM.pdf]

Changes in five cerebrospinal fluid and three plasma Alzheimer's disease biomarkers across increasing brain amyloid-beta and tau pathology burden

Andrea Mastrangelo[1,2], Simone Baiardi[2], Edoardo Ruggeri[2], Giuseppe Mario Bentivenga[1], Claudia Marina Vargiu[2], Angela Mammana[2], Marco Sbriccoli[3], Barbara Polischi[2], Benedetta Carlà[2], Sabina Capellari[1,2], Piero Parchi[1,2]

[1]Department of Biomedical and Neuromotor Sciences, University of Bologna, Bologna, Italy

[2]IRCCS, Istituto delle Scienze Neurologiche di Bologna, Bologna, Italy

[3]Department of Neuroscience, Istituto Superiore di Sanità, Rome, Italy

Correspondence to Piero Parchi:

Email: piero.parchi@unibo.it

Phone number: +39-051496674

SUPPLEMENTARY TABLES

Supplementary Table 1 - Demographic and basic neuropathological features in the non-prion subgroup with complete CSF biomarker availability

	Non-prion CSF cohort with complete CSF biomarker availability (n=61)
**Age at CSF sampling[a]**	74.1 (63.1-78.6)
**Age at death[a]**	74.6 (66.0-80.0)
**Females**	35 (57.4)
***APOE* ε4 carriers**	10 (16.4)
**Time CSF sampling-death[b]**	1.0 (0.2-7.8)
**Thal phase**	
0	17 (27.9)
1-2	15 (24.6)
3	16 (26.2)
4-5	13 (21.3)
**Braak stage**	
0	24 (39.3)
1-2	23 (37.7)
3-4	12 (19.7)
5-6	2 (3.3)
**ADNC level**	
Not AD	17 (27.9)
Low	30 (49.2)
Intermediate	12 (19.7)
High	2 (3.3)
**Aβ score quartiles**	
Reference	15 (24.6)
1st quartile	14 (22.9)
2nd quartile	14 (22.9)
3rd quartile	8 (13.1)
4th quartile	10 (16.4)
**Tau score quartiles**	
Reference	14 (22.9)
1st quartile	17 (27.9)
2nd quartile	10 (16.4)
3rd quartile	13 (21.3)
4th quartile	7 (11.5)

a: expressed in years; b: expressed in months.

Continuous variables are expressed as median (interquartile range). Categorical variables are expressed as number (%). Abbreviations: AD, Alzheimer's disease; ADNC, Alzheimer's disease neuropathologic change; CSF, cerebrospinal fluid

Supplementary Table 2 - Details on primary neuropathological diagnoses of participants included in the study

	Entire cohort (N=250)	CSF cohort (n=230)	Plasma cohort (n=101)
**Prion disease**	**162**	**159**	**41**
sCJD MM(V)1	63	61	19
sCJD MM(V1) + 2C	28	27	4
sCJD VV2	19	19	6
sCJD MV2K	17	17	4
sCJD MV2K+2C	3	3	0
sCJD MM(V)2C	4	4	0
sCJD MM(V)2C+1	5	5	1
sCJD MM2T+2C	1	1	0
sCJD VV1	2	2	1
sCJD VM1	1	1	0
VPSPr	1	1	1
gCJD, M1	15	15	4
gCJD, atypical	1	1	1
FFI	2	2	0
**Non-prion disease**	**88**	**71**	**60**
AD, High ADNC	6	4	6
AD, Intermediate ADNC	9	9	5
AD, Low ADNC	10	8	8
LBD, neocortical subtype	7	4	5
FTLD, TDP-43	5	3	5
FTLD, tau	2	2	0
MSA	7	5	5
PART	3	2	1
Encephalitis	6	5	5
IRIS encephalitis	2	2	2
Vascular disease[a]	10	8	8
Neoplastic disease[b]	6	5	2
Wernicke's encephalopathy	4	3	1
Other[c]	11	11	7

a: including subcortical ischemic vascular disease (n=6), cerebral infarction (n=3), haemorrhagic subacute encephalopathy (n=1); b: including primary central nervous system lymphoma (n=2), intravascular lymphoma (n=2), neoplastic meningitis (n=2); c: including lymphocytic meningitis (n=2), progressive multifocal leukoencephalopathy (n=1), not otherwise specified leukoencephalopathy (n=1), non-specific neuropathologic changes (n=7)

Abbreviations: AD, Alzheimer's disease; ADNC, Alzheimer's disease neuropathologic change; CSF, cerebrospinal fluid; FFI, fatal familial insomnia; FTLD, frontotemporal lobar degeneration; gCJD, genetic Creutzfeldt-Jakob disease; IRIS, immune reconstitution inflammatory syndrome; LBD, Lewy Body disease; MSA, multiple system atrophy; PART, primary age-related tauopathy; sCJD, sporadic Creutzfeldt-Jakob disease; VPSPr, variably protease-sensitive prionopathy

Supplementary Table 3 - Associations of biofluid biomarkers with continuous values of Aβ and tau pathology scores

		**Aβ score**	**Tau score**	**Combined model**	
				**Aβ score**	**Tau score**
CSF cohort					
**CSF $A\beta_{42}/A\beta_{40}$**	**β coefficient**	-0.01 (-0.02 to -0.01)	-0.02 (-0.02 to -0.02)	-0.01 (-0.01 to -0.01)	-0.004 (-0.01 to -0.001)
	**p value**	**<0.001**	**<0.001**	**<0.001**	**0.021**
Non-prion CSF cohort					
**CSF $A\beta_{42}/A\beta_{40}$**	**β coefficient**	-0.02 (-0.02 to -0.01)	-0.02 (-0.03 to -0.01)	-0.01 (-0.02 to -0.01)	-0.001 (-0.01 to 0.003)
	**p value**	**<0.001**	**<0.001**	**<0.001**	0.495
**CSF p-tau_{181}**	**β coefficient**	0.02 (0.01 to 0.03)	0.04 (0.03 to 0.05)	0.01 (-0.002 to 0.02)	0.03 (0.01 to 0.05)
	**p value**	**<0.001**	**<0.001**	0.098	**0.002**
**CSF $A\beta_{42}$/p-tau_{181}**	**β coefficient**	-0.03 (-0.04 to -0.03)	-0.06 (-0.07 to -0.05)	-0.01 (-0.02 to -0.004)	-0.04 (-0.06 to -0.03)
	**p value**	**<0.001**	**<0.001**	**0.006**	**<0.001**
**CSF p-tau_{217}**	**β coefficient**	0.04 (0.03 to 0.05)	0.08 (0.06 to 0.10)	0.02 (0.005 to 0.03)	0.05 (0.02 to 0.08)
	**p value**	**<0.001**	**<0.001**	**0.008**	**<0.001**
**CSF $A\beta_{42}$/p-tau_{217}**	**β coefficient**	-0.05 (-0.06 to -0.04)	-0.09 (-0.11 to -0.07)	-0.03 (-0.04 to -0.01)	-0.05 (-0.08 to -0.03)
	**p value**	**<0.001**	**<0.001**	**<0.001**	**<0.001**
Plasma cohort					
**Plasma $A\beta_{42}/A\beta_{40}$**	**β coefficient**	-0.001 (-0.003 to 0.001)	0.001 (-0.002 to 0.005)	-0.003 (-0.006 to -0.0004)	0.005 (0.001 to 0.01)
	**p value**	0.234	0.533	**0.026**	**0.047**
Non-prion plasma cohort					
**Plasma $A\beta_{42}/A\beta_{40}$**	**β coefficient**	-0.0005 (-0.003 to 0.002)	0.002 (-0.002 to 0.006)	-0.003 (-0.008 to 0.0009)	0.006 (-0.0005 to 0.01)
	**p value**	0.702	0.333	0.116	0.071
**Plasma p-tau_{217}**	**β coefficient**	0.02 (0.01 to 0.03)	0.03 (0.02 to 0.04)	0.007 (-0.01 to 0.02)	0.02 (0.004 to 0.04)
	**p value**	**<0.001**	**<0.001**	0.303	**0.019**
**Plasma p-$tau_{217}/A\beta_{42}$**	**β coefficient**	0.02 (0.01 to 0.03)	0.04 (0.02 to 0.05)	0.004 (-0.01 to 0.02)	0.03 (0.01 to 0.05)
	**p value**	**<0.001**	**<0.001**	0.558	**<0.001**

Results are derived from multivariable linear regression models with log-transformed biomarker values as dependent variables and continuous values of Aβ and tau score, both separately and as simultaneous predictors as independent variables. Separate models were fitted for each biomarker-pathology association. All models were adjusted for age at CSF/plasma sampling, sex and time between CSF/plasma sampling and death; models including participants with prion disease were additionally adjusted for prion disease status. Analyses on CSF p-tau_{217} and $A\beta_{42}$/p-tau_{217} were performed in a subgroup (n=61). β coefficients are reported as value (95% confidence interval). P values of statistically significant associations are shown in bold. Abbreviations: CSF, cerebrospinal fluid

Supplementary Table 4 - Association of Aβ and tau pathology score levels with changes in CSF and plasma biomarkers

Aβ pathology score					
	**β coefficient**	**p value**		**β coefficient**	**p value**
**CSF cohort (n=230)**			**Plasma cohort (n=101)**		
CSF $A\beta_{42}/A\beta_{40}$			***Plasma $A\beta_{42}/A\beta_{40}$***		
1st quartile	-0.02 (-0.10 to 0.05)	0.567	*1st quartile*	-0.007 (-0.12 to 0.11)	0.896
2nd quartile	**-0.11 (-0.19 to -0.04)**	**0.001**	*2nd quartile*	-0.04 (-0.15 to 0.08)	0.527
3rd quartile	**-0.42 (-0.51 to -0.34)**	**<0.001**	*3rd quartile*	**-0.14 (-0.26 to -0.02)**	**0.025**
4th quartile	**-0.62 (-0.71 to -0.54)**	**<0.001**	*4th quartile*	-0.02 (-0.14 to 0.09)	0.670
**Non-prion CSF cohort (n=71)**			**Non-prion plasma cohort (n=60)**		
CSF $A\beta_{42}/A\beta_{40}$			***Plasma $A\beta_{42}/A\beta_{40}$***		
1st quartile	-0.05 (-0.18 to 0.09)	0.488	*1st quartile*	-0.07 (-0.25 to 0.10)	0.390
2nd quartile	**-0.23 (-0.36 to -0.09)**	**0.001**	*2nd quartile*	-0.02 (-0.19 to 0.15)	0.849
3rd quartile	**-0.57 (-0.73 to -0.41)**	**<0.001**	*3rd quartile*	0.03 (-0.17 to 0.23)	0.760
4th quartile	**-0.80 (-0.95 to -0.65)**	**<0.001**	*4th quartile*	-0.06 (-0.24 to 0.12)	0.504
CSF p-tau_{181}			***Plasma p-tau_{217}***		
1st quartile	0.03 (-0.38 to 0.44)	0.886	*1st quartile*	-0.13 (-0.70 to 0.44)	0.653
2nd quartile	0.23 (-0.18 to 0.65)	0.261	*2nd quartile*	-0.06 (-0.63 to 0.51)	0.828
3rd quartile	**0.64 (0.15 to 1.13)**	**0.011**	*3rd quartile*	0.42 (-0.25 to 1.08)	0.213
4th quartile	**1.20 (0.73 to 1.67)**	**<0.001**	*4th quartile*	**0.86 (0.26 to 1.46)**	**0.005**
CSF $A\beta_{42}$/p-tau_{181}			***Plasma p-$tau_{217}/A\beta_{42}$***		
1st quartile	-0.05 (-0.50 to 0.40)	0.830	*1st quartile*	-0.07 (-0.64 to 0.50)	0.794
2nd quartile	-0.36 (-0.82 to 0.09)	0.113	*2nd quartile*	-0.06 (-0.63 to 0.50)	0.822
3rd quartile	**-1.33 (-1.86 to -0.79)**	**<0.001**	*3rd quartile*	0.32 (-0.34 to 0.98)	0.333
4th quartile	**-1.66 (-2.19 to -1.15)**	**<0.001**	*4th quartile*	**0.91 (0.32 to 1.51)**	**0.003**
	CSF p-tau_{217}				
1st quartile	0.23 (-0.30 to 0.77)	0.387			
2nd quartile	0.50 (-0.05 to 1.04)	0.074			
3rd quartile	**1.19 (0.52 to 1.86)**	**<0.001**			
4th quartile	**2.18 (1.53 to 2.82)**	**<0.001**			
CSF $A\beta_{42}$/p-tau_{217}					
1st quartile	-0.19 (-0.73 to 0.35)	0.478			
2nd quartile	**-0.65 (-1.20 to -0.11)**	**0.020**			
3rd quartile	**-1.55 (-2.22 to -0.87)**	**<0.001**			
4th quartile	**-2.57 (-3.21 to -1.92)**	**<0.001**			
**Tau pathology score**					
**Non-prion CSF cohort (n=71)**			**Non-prion plasma cohort (n=60)**		
CSF p-tau_{181}			***Plasma p-tau_{217}***		
1st quartile	-0.15 (-0.55 to 0.26)	0.472	*1st quartile*	-0.29 (-0.81 to 0.23)	0.271
2nd quartile	0.18 (-0.30 to 0.67)	0.448	*2nd quartile*	0.15 (-0.51 to 0.82)	0.641
3rd quartile	**0.82 (0.32 to 1.32)**	**0.001**	*3rd quartile*	-0.27 (-0.95 to 0.41)	0.430
4th quartile	**1.12 (0.63 to 1.62)**	**<0.001**	*4th quartile*	**0.68 (0.06 to 1.29)**	**0.032**
CSF $A\beta_{42}$/p-tau_{181}			***Plasma p-$tau_{217}/A\beta_{42}$***		
1st quartile	-0.13 (-0.53 to 0.26)	0.501	*1st quartile*	-0.09 (-0.58 to 0.39)	0.698
2nd quartile	-0.36 (-0.83 to 0.11)	0.128	*2nd quartile*	0.11 (-0.51 to 0.73)	0.718
3rd quartile	**-1.16 (-1.64 to -0.67)**	**<0.001**	*3rd quartile*	-0.03 (-0.66 to 0.60)	0.924
4th quartile	**-2.14 (-2.62 to -1.65)**	**<0.001**	*4th quartile*	**1.05 (0.48 to 1.62)**	**<0.001**
CSF p-tau_{217}					
1st quartile	-0.27 (-0.85 to 0.30)	0.348			
2nd quartile	0.03 (-0.65 to 0.72)	0.921			
3rd quartile	**1.12 (0.41 to 1.83)**	**0.002**			
4th quartile	**1.90 (1.11 to 2.69)**	**<0.001**			
CSF $A\beta_{42}$/p-tau_{217}					
1st quartile	-0.02 (-0.60 to 0.54)	0.928			
2nd quartile	-0.21 (-0.89 to 0.46)	0.529			
3rd quartile	**-1.44 (-2.14 to -0.74)**	**<0.001**			
4th quartile	**-2.71 (-3.49 to -1.93)**	**<0.001**			

Results are derived from multivariable linear regression models with log-transformed biomarker values as dependent variables and categorical quartiles of Aβ or tau pathology scores as independent variables. Separate models were fitted for each biomarker-pathology association. All models were adjusted for age at CSF/plasma sampling, sex and time between CSF/plasma sampling and death; models including participants with prion disease were additionally adjusted for prion disease status. In each model, the reference category consisted of participants with a corresponding pathology score of 0. β coefficients are reported as value (95% confidence interval). Analyses on CSF p-tau_{217} and $A\beta_{42}$/p-tau_{217} were performed in a subgroup (n=61). β coefficients and p values corresponding to pathology levels showing statistically significant differences from the reference group are indicated in bold. Abbreviations: CSF, cerebrospinal fluid

Supplementary Table 5 - Association of Aβ and tau pathology score levels with changes in CSF biomarkers in the subgroup of non-prion participants with complete CSF biomarker availability

Aβ pathology score		
	**β coefficient**	**p value**
**Non-prion CSF cohort (n=61)**		
CSF $A\beta_{42}/A\beta_{40}$		
1st quartile	-0.05 (-0.20 to 0.10)	0.528
2nd quartile	**-0.25 (-0.41 to -0.10)**	**0.002**
3rd quartile	**-0.56 (-0.75 to -0.37)**	**<0.001**
4th quartile	**-0.80 (-0.99 to -0.62)**	**<0.001**
CSF p-tau$_{181}$		
1st quartile	0.12 (-0.32 to 0.57)	0.573
2nd quartile	0.36 (-0.09 to 0.81)	0.118
3rd quartile	**0.65 (0.09 to 1.20)**	**0.023**
4th quartile	**1.21 (0.68 to 1.74)**	**<0.001**
CSF $A\beta_{42}$/p-tau$_{181}$		
1st quartile	-0.08 (-0.52 to 0.35)	0.706
2nd quartile	**-0.51 (-0.96 to -0.07)**	**0.024**
3rd quartile	**-1.01 (-1.55 to -0.46)**	**<0.001**
4th quartile	**-1.60 (-2.13 to -1.08)**	**<0.001**
**Tau pathology score**		
**Non-prion CSF cohort (n=61)**		
CSF p-tau$_{181}$		
1st quartile	-0.20 (-0.64 to 0.24)	0.369
2nd quartile	0.03 (-0.49 to 0.55)	0.903
3rd quartile	**0.82 (0.29 to 1.36)**	**0.003**
4th quartile	**1.05 (0.45 to 1.66)**	**<0.001**
CSF $A\beta_{42}$/p-tau$_{181}$		
1st quartile	-0.10 (-0.52 to 0.32)	0.637
2nd quartile	-0.21 (-0.72 to 0.29)	0.402
3rd quartile	**-1.14 (-1.66 to -0.62)**	**<0.001**
4th quartile	**-1.86 (-2.45 to -1.28)**	**<0.001**

Results are derived from multivariable linear regression models with log-transformed biomarker values as dependent variables and categorical quartiles of Aβ or tau pathology scores as independent variables. Separate models were fitted for each biomarker-pathology association. All models were adjusted for age at CSF sampling, sex and time between CSF sampling and death. In each model, the reference category consisted of participants with a corresponding pathology score of 0. β coefficients are reported as value (95% confidence interval). β coefficients and p values corresponding to pathology levels showing statistically significant differences from the reference group are indicated in bold. Abbreviations: CSF, cerebrospinal fluid

Supplementary Table 6 - Association of tau pathology score levels with changes in CSF and plasma biomarkers in the subgroup of participants without PART

Tau pathology score					
**Non-prion CSF cohort, non-PART (n=65)**			**Non-prion plasma cohort, non-PART (n=57)**		
CSF p-tau$_{181}$			***Plasma p-tau$_{217}$***		
1st quartile	-0.15 (-0.55 to 0.25)	0.451	*1st quartile*	-0.27 (-0.81 to 0.27)	0.319
2nd quartile	0.34 (-0.21 to 0.90)	0.220	*2nd quartile*	0.15 (-0.61 to 0.91)	0.697
3rd quartile	**0.84 (0.34 to 1.34)**	**0.001**	*3rd quartile*	-0.27 (-0.98 to 0.43)	0.443
4th quartile	**1.14 (0.65 to 1.63)**	**<0.001**	*4th quartile*	**0.68 (0.04 to 1.32)**	**0.037**
CSF Aβ$_{42}$/p-tau$_{181}$			***Plasma p-tau$_{217}$/Aβ$_{42}$***		
1st quartile	-0.13 (-0.53 to 0.26)	0.504	*1st quartile*	-0.16 (-0.63 to 0.30)	0.494
2nd quartile	-0.38 (-0.93 to 0.18)	0.181	*2nd quartile*	0.16 (-0.49 to 0.82)	0.614
3rd quartile	**-1.21 (-1.71 to -0.71)**	**<0.001**	*3rd quartile*	0.02 (-0.59 to 0.63)	0.944
4th quartile	**-2.15 (-2.64 to -1.65)**	**<0.001**	*4th quartile*	**1.09 (0.53 to 1.64)**	**<0.001**
CSF p-tau$_{217}$					
1st quartile	-0.29 (-0.88 to 0.30)	0.323			
2nd quartile	0.26 (-0.52 to 1.06)	0.503			
3rd quartile	**1.13 (0.40 to 1.86)**	**0.003**			
4th quartile	**1.89 (1.09 to 2.69)**	**<0.001**			
CSF Aβ$_{42}$/p-tau$_{217}$					
1st quartile	-0.02 (-0.60 to 0.57)	0.949			
2nd quartile	-0.31 (-1.09 to 0.48)	0.437			
3rd quartile	**-1.49 (-2.22 to -0.76)**	**<0.001**			
4th quartile	**-2.69 (-3.49 to -1.89)**	**<0.001**			

Results are derived from multivariable linear regression models with log-transformed biomarker values as dependent variables and categorical quartiles of tau pathology scores as independent variables. Separate models were fitted for each biomarker. All models were adjusted for age at CSF/plasma sampling, sex and time between CSF/plasma sampling and death. In each model, the reference category consisted of participants with a tau pathology score of 0. β coefficients are reported as value (95% confidence interval). Analyses on CSF p-tau$_{217}$ and Aβ$_{42}$/p-tau$_{217}$ were performed in a subgroup (n=56). β coefficients and p values corresponding to pathology levels showing statistically significant differences from the reference group are indicated in bold. Abbreviations: CSF, cerebrospinal fluid; PART, primary age-related tauopathy

Supplementary Table 7 - Association of tau pathology score levels with changes in CSF and plasma biomarkers after correction for Aβ pathology score

Tau pathology score					
Non-prion CSF cohort (n=71)			Non-prion plasma cohort (n=60)		
CSF p-tau$_{181}$			*Plasma p-tau$_{217}$*		
1st quartile	-0.17 (-0.57 to 0.22)	0.388	*1st quartile*	-0.34 (-0.84 to 0.17)	0.185
2nd quartile	0.11 (-0.36 to 0.59)	0.632	*2nd quartile*	0.03 (-0.61 to 0.67)	0.922
3rd quartile	**0.58 (0.02 to 1.13)**	**0.041**	*3rd quartile*	-0.67 (-1.40 to 0.06)	0.071
4th quartile	**0.72 (0.07 to 1.37)**	**0.031**	*4th quartile*	0.08 (-0.68 to 0.85)	0.822
CSF Aβ$_{42}$/p-tau$_{181}$			*Plasma p-tau$_{217}$/Aβ$_{42}$*		
1st quartile	-0.10 (-0.47 to 0.28)	0.606	*1st quartile*	-0.12 (-0.60 to 0.36)	0.607
2nd quartile	-0.27 (-0.72 to 0.18)	0.242	*2nd quartile*	0.03 (-0.58 to 0.65)	0.918
3rd quartile	**-0.82 (-1.35 to -0.30)**	**0.002**	*3rd quartile*	-0.29 (-0.99 to 0.41)	0.410
4th quartile	**-1.58 (-2.20 to -0.96)**	**<0.001**	*4th quartile*	0.67 (-0.06 to 1.40)	0.073
CSF p-tau$_{217}$					
1st quartile	-0.34 (-0.87 to 0.19)	0.204			
2nd quartile	-0.15 (-0.79 to 0.48)	0.625			
3rd quartile	0.56 (-0.16 to 1.29)	0.125			
4th quartile	**0.92 (0.003 to 1.85)**	**0.049**			
CSF Aβ$_{42}$/p-tau$_{217}$					
1st quartile	0.05 (-0.43 to 0.54)	0.815			
2nd quartile	0.02 (-0.56 to 0.61)	0.937			
3rd quartile	**-0.74 (-1.41 to -0.07)**	**0.030**			
4th quartile	**-1.49 (-2.34 to -0.64)**	**<0.001**			

Results are derived from multivariable linear regression models with log-transformed biomarker values as dependent variables and categorical quartiles of tau pathology scores as independent variables. Separate models were fitted for each biomarker. All models were adjusted for age at CSF/plasma sampling, sex, time between CSF/plasma sampling and death and continuous Aβ pathology score. In each model, the reference category consisted of participants with a tau pathology score of 0. β coefficients are reported as value (95% confidence interval). Analyses on CSF p-tau$_{217}$ and Aβ$_{42}$/p-tau$_{217}$ were performed in a subgroup (n=61). β coefficients and p values corresponding to pathology levels showing statistically significant differences from the reference group are indicated in bold. Abbreviations: CSF, cerebrospinal fluid

Supplementary Table 8 - Association of Thal phase levels with changes in CSF and plasma biomarkers

Thal phase					
	**β coefficient**	**p value**		**β coefficient**	**p value**
**CSF cohort (n=230)**			**Plasma cohort (n=101)**		
CSF $A\beta_{42}/A\beta_{40}$			***Plasma $A\beta_{42}/A\beta_{40}$***		
1-2	**-0.07 (-0.14 to -0.01)**	**0.026**	*1-2*	-0.02 (-0.12 to 0.08)	0.706
3	**-0.31 (-0.39 to -0.24)**	**<0.001**	*3*	-0.05 (-0.17 to 0.06)	0.372
4-5	**-0.63 (-0.72 to -0.55)**	**<0.001**	*4-5*	-0.10 (-0.21 to 0.01)	0.077
**Non-prion CSF cohort (n=71)**			**Non-prion plasma cohort (n=60)**		
CSF $A\beta_{42}/A\beta_{40}$			***Plasma $A\beta_{42}/A\beta_{40}$***		
1-2	0.009 (-0.12 to 0.13)	0.885	*1-2*	-0.04 (-0.20 to 0.12)	0.640
3	**-0.34 (-0.48 to -0.21)**	**<0.001**	*3*	-0.01 (-0.20 to 0.17)	0.880
4-5	**-0.71 (-0.84 to -0.57)**	**<0.001**	*4-5*	-0.08 (-0.25 to 0.09)	0.349
CSF p-tau_{181}			***Plasma p-tau_{217}***		
1-2	-0.02 (-0.41 to 0.36)	0.899	*1-2*	-0.13 (-0.66 to 0.41)	0.634
3	0.32 (-0.10 to 0.74)	0.137	*3*	0.06 (-0.56 to 0.67)	0.853
4-5	**1.01 (0.59 to 1.43)**	**<0.001**	*4-5*	**0.69 (0.12 to 1.27)**	**0.019**
CSF $A\beta_{42}$/p-tau_{181}			***Plasma p-$tau_{217}/A\beta_{42}$***		
1-2	0.07 (-0.33 to 0.48)	0.713	*1-2*	-0.07 (-0.60 to 0.47)	0.797
3	**-0.59 (-1.03 to -0.15)**	**0.010**	*3*	0.13 (-0.49 to 0.74)	0.677
4-5	**-1.60 (-2.03 to -1.16)**	**<0.001**	*4-5*	**0.75 (0.17 to 1.32)**	**0.012**
CSF p-tau_{217}					
1-2	0.16 (-0.34 to 0.67)	0.520			
3	**0.58 (0.04 to 1.13)**	**0.035**			
4-5	**1.96 (1.38 to 2.55)**	**<0.001**			
CSF $A\beta_{42}$/p-tau_{217}					
1-2	-0.09 (-0.60 to 0.42)	0.716			
3	**-0.81 (-1.36 to -0.27)**	**0.004**			
4-5	**-2.35 (-2.93 to -1.76)**	**<0.001**			

Results are derived from multivariable linear regression models with log-transformed biomarker values as dependent variables and categorical Thal phases (1-2, 3 and 4-5) as independent variables. Separate models were fitted for each biomarker. All models were adjusted for age at CSF/plasma sampling, sex and time between CSF/plasma sampling and death; models including participants with prion disease were additionally adjusted for prion disease status. In each model, the reference category consisted of participants with Thal phase of 0. Analyses on CSF p-tau_{217} and $A\beta_{42}$/p-tau_{217} were performed in a subgroup (n=61). β coefficients are reported as value (95% confidence interval). β coefficients and p values corresponding to pathology levels showing statistically significant differences from the reference group are indicated in bold. Abbreviations: CSF, cerebrospinal fluid

Supplementary Table 9 - Association of Braak stage levels with changes in CSF and plasma biomarkers

Braak stage					
	**β coefficient**	**p value**		**β coefficient**	**p value**
**Non-prion CSF cohort (n=71)**			**Non-prion plasma cohort (n=60)**		
CSF p-tau$_{181}$			***Plasma p-tau$_{217}$***		
1-2	0.28 (-0.06 to 0.61)	0.106	*1-2*	-0.14 (-0.58 to 0.29)	0.515
3-4	**1.19 (0.76 to 1.61)**	**<0.001**	*3-4*	0.17 (-0.38 to 0.71)	0.541
5-6	**1.38 (0.84 to 1.93)**	**<0.001**	*5-6*	**1.20 (0.64 to 1.76)**	**<0.001**
CSF Aβ$_{42}$/p-tau$_{181}$			***Plasma p-tau$_{217}$/Aβ$_{42}$***		
1-2	**-0.37 (-0.72 to -0.03)**	**0.034**	*1-2*	0.25 (-0.16 to 0.66)	0.224
3-4	**-1.74 (-2.17 to -1.31)**	**<0.001**	*3-4*	**0.69 (0.17 to 1.20)**	**0.009**
5-6	**-2.09 (-2.65 to -1.54)**	**<0.001**	*5-6*	**1.56 (1.04 to 2.09)**	**<0.001**
CSF p-tau$_{217}$					
1-2	0.37 (-0.09 to 0.84)	0.115			
3-4	**1.91 (1.31 to 2.51)**	**<0.001**			
5-6	**2.69 (1.65 to 3.73)**	**<0.001**			
CSF Aβ$_{42}$/p-tau$_{217}$					
1-2	-0.45 (-0.93 to 0.03)	0.065			
3-4	**-2.29 (-2.90 to -1.67)**	**<0.001**			
5-6	**-3.05 (-4.12 to -1.98)**	**<0.001**			
**Non-prion CSF cohort, non-PART (n=65)**			**Non-prion plasma cohort, non-PART (n=57)**		
CSF p-tau$_{181}$			***Plasma p-tau$_{217}$***		
1-2	0.32 (-0.03 to 0.67)	0.074	*1-2*	-0.17 (-0.63 to 0.29)	0.455
3-4	**1.23 (0.82 to 1.64)**	**<0.001**	*3-4*	0.16 (-0.40 to 0.71)	0.573
5-6	**1.41 (0.88 to 1.94)**	**<0.001**	*5-6*	**1.19 (0.62 to 1.77)**	**<0.001**
CSF Aβ$_{42}$/p-tau$_{181}$			***Plasma p-tau$_{217}$/Aβ$_{42}$***		
1-2	**-0.39 (-0.76 to -0.02)**	**0.039**	*1-2*	0.17 (-0.23 to 0.58)	0.402
3-4	**-1.76 (-2.20 to -1.32)**	**<0.001**	*3-4*	**0.70 (0.21 to 1.19)**	**0.006**
5-6	**-2.11 (-2.67 to -1.54)**	**<0.001**	*5-6*	**1.57 (1.06 to 2.08)**	**<0.001**
CSF p-tau$_{217}$					
1-2	0.45 (-0.04 to 0.94)	0.072			
3-4	**1.93 (1.33 to 2.53)**	**<0.001**			
5-6	**2.74 (1.70 to 3.79)**	**<0.001**			
CSF Aβ$_{42}$/p-tau$_{217}$					
1-2	-0.50 (-1.02 to 0.01)	0.056			
3-4	**-2.29 (-2.92 to -1.65)**	**<0.001**			
5-6	**-3.06 (-4.16 to -1.95)**	**<0.001**			

Results are derived from multivariable linear regression models with log-transformed biomarker values as dependent variables and categorical Braak stages (1-2, 3-4 and 5-6) as independent variables. Separate models were fitted for each biomarker. All models were adjusted for age at CSF/plasma sampling, sex and time between CSF/plasma sampling and death; models including participants with prion disease were additionally adjusted for prion disease status. In each model, the reference category consisted of participants with Braak stage of 0. Analyses were repeated after exclusion of subjects with PART (i.e., showing tau pathology without Aβ deposits). Analyses on CSF p-tau$_{217}$ and Aβ$_{42}$/p-tau$_{217}$ were performed in a subgroup (n=61 including PART, n=56 excluding PART). β coefficients are reported as value (95% confidence interval). β coefficients and p values corresponding to pathology levels showing statistically significant differences from the reference group are indicated in bold. Abbreviations: CSF, cerebrospinal fluid

Supplementary Table 10 - Association of ADNC levels with changes in CSF and plasma biomarkers

ADNC levels					
	**β coefficient**	**p value**		**β coefficient**	**p value**
**CSF cohort (n=230)**			**Plasma cohort (n=101)**		
CSF $A\beta_{42}/A\beta_{40}$			***Plasma $A\beta_{42}/A\beta_{40}$***		
Low	**-0.18 (-0.25 to -0.12)**	**<0.001**	*Low*	-0.05 (-0.15 to 0.04)	0.254
Intermediate	**-0.67 (-0.77 to -0.56)**	**<0.001**	*Intermediate*	-0.09 (-0.21 to 0.04)	0.168
High	**-0.62 (-0.83 to -0.41)**	**<0.001**	*High*	0.06 (-0.10 to 0.21)	0.477
**Non-prion CSF cohort (n=71)**			**Non-prion plasma cohort (n=60)**		
CSF $A\beta_{42}/A\beta_{40}$			***Plasma $A\beta_{42}/A\beta_{40}$***		
Low	**-0.15 (-0.28 to -0.02)**	**0.025**	*Low*	-0.06 (-0.21 to 0.08)	0.385
Intermediate	**-0.73 (-0.91 to -0.55)**	**<0.001**	*Intermediate*	-0.06 (-0.24 to 0.13)	0.545
High	**-0.61 (-0.84 to -0.38)**	**<0.001**	*High*	0.07 (-0.13 to 0.26)	0.492
CSF $p\text{-}tau_{181}$			***Plasma $p\text{-}tau_{217}$***		
Low	0.09 (-0.23 to 0.41)	0.561	*Low*	-0.08 (-0.53 to 0.37)	0.718
Intermediate	**1.07 (0.64 to 1.51)**	**<0.001**	*Intermediate*	0.23 (-0.35 to 0.81)	0.435
High	**1.28 (0.72 to 1.84)**	**<0.001**	*High*	**1.22 (0.62 to 1.83)**	**<0.001**
CSF $A\beta_{42}/p\text{-}tau_{181}$			***Plasma $p\text{-}tau_{217}/A\beta_{42}$***		
Low	-0.18 (-0.51 to 0.15)	0.287	*Low*	-0.05 (-0.48 to 0.38)	0.811
Intermediate	**-1.62 (-2.07 to -1.18)**	**<0.001**	*Intermediate*	0.44 (-0.11 to 0.99)	0.117
High	**-1.99 (-2.57 to -1.42)**	**<0.001**	*High*	**1.34 (0.76 to 1.92)**	**<0.001**
CSF $p\text{-}tau_{217}$					
Low	0.35 (-0.07 to 0.77)	0.104			
Intermediate	**1.91 (1.32 to 2.49)**	**<0.001**			
High	**2.70 (1.66 to 3.74)**	**<0.001**			
CSF $A\beta_{42}/p\text{-}tau_{217}$					
Low	-0.41 (-0.85 to 0.02)	0.065			
Intermediate	**-2.27 (-2.88 to -1.67)**	**<0.001**			
High	**-3.05 (-4.12 to -1.98)**	**<0.001**			

Results are derived from multivariable linear regression models with log-transformed biomarker values as dependent variables and categorical ADNC levels (Low, Intermediate, High) as independent variables. Separate models were fitted for each biomarker. All models were adjusted for age at CSF/plasma sampling, sex and time between CSF/plasma sampling and death; models including participants with prion disease were additionally adjusted for prion disease status. In each model, the reference category consisted of participants classified as not AD. Analyses on CSF p-tau_{217} and $A\beta_{42}$/p-tau_{217} were performed in a subgroup (n=61). β coefficients are reported as value (95% confidence interval). β coefficients and p values corresponding to pathology levels showing statistically significant differences from the reference group are indicated in bold. Abbreviations: ADNC, Alzheimer's disease neuropathologic change; CSF, cerebrospinal fluid

Supplementary Table 11 – Discriminatory performance of CSF and plasma biomarkers across increasing levels of Aβ pathology burden

	AUC	Maximized Youden-index cut-off			≃95% specificity cut-off
		Value	Sensitivity	Specificity	Value
**CSF cohort (n=230)**					
**CSF $A\beta_{42}/A\beta_{40}$**					
>0	0.829 (0.777-0.881)	<0.080	69.4 (61.8-76.0)	85.7 (75.7-92.0)	<0.074
>8	0.886 (0.843-0.929)	<0.074	69.9 (60.9-77.6)	93.2 (87.1-96.5)	<0.071
>16	0.937 (0.903-0.971)	<0.069	78.6 (68.6-86.0)	96.6 (92.2-98.5)	<0.070
>24	0.966 (0.943-0.989)	<0.066	91.5 (81.6-96.3)	94.7 (90.3-97.2)	<0.066
>32	0.950 (0.921-0.979)	<0.066	95.1 (83.9-99.1)	87.3 (81.8-91.3)	<0.052
>40	0.935 (0.895-0.974)	<0.066	95.6 (79.0-99.8)	80.2 (74.2-85.0)	<0.046
**Non-prion CSF cohort (n=71)**					
**CSF $A\beta_{42}/A\beta_{40}$**					
>0	0.873 (0.792-0.954)	<0.078	74.1 (61.1-83.9)	100 (81.6-100)	<0.078
>8	0.939 (0.879-0.999)	<0.071	84.6 (70.3-92.7)	96.9 (84.3-99.8)	<0.074
>16	0.984 (0.964-1.000)	<0.070	93.7 (79.8-98.9)	92.3 (79.7-97.3)	<0.069
>24	0.964 (0.926-1.000)	<0.054	88.0 (70.0-95.8)	95.6 (85.5-99.2)	<0.054
>32	0.977 (0.949-1.000)	<0.054	100 (83.2-100)	90.4 (79.4-95.8)	<0.052
>40	0.952 (0.904-0.999)	<0.054	100 (77.2-100)	81.0 (69.1-89.1)	<0.046
**CSF p-tau_{181}**					
>0	0.710 (0.573-0.846)	>36.5	57.4 (44.2-69.7)	82.3 (59.0-93.8)	>79.0
>8	0.782 (0.675-0.890)	>37.5	69.2 (53.6-81.4)	81.2 (64.7-91.1)	>79.0
>16	0.833 (0.737-0.929)	>37.5	81.2 (64.7-91.1)	82.0 (67.3-91.0)	>79.0
>24	0.819 (0.720-0.918)	>37.5	84.0 (65.3-93.6)	73.9 (59.7-84.4)	>103.5
>32	0.889 (0.810-0.968)	>46.5	89.5 (68.6-98.1)	80.8 (68.1-89.2)	>103.5
>40	0.887 (0.807-0.967)	>46.5	92.3 (66.7-99.6)	74.1 (61.6-83.6)	>103.5
**CSF $A\beta_{42}$/p-tau_{181}**					
>0	0.791 (0.678-0.903)	<13.36	74.1 (61.1-83.9)	82.3 (59.0-93.8)	<9.31
>8	0.835 (0.745-0.925)	<14.91	87.2 (73.3-94.4)	62.5 (45.2-77.1)	<7.99
>16	0.909 (0.846-0.973)	<13.36	96.9 (84.3-99.8)	69.2 (53.6-81.4)	<7.99
>24	0.914 (0.848-0.980)	<8.50	80.0 (60.9-91.1)	91.3 (79.7-96.6)	<6.68
>32	0.954 (0.907-1.000)	<8.50	94.7 (75.4-99.7)	88.5 (77.0-94.6)	<6.68
>40	0.900 (0.822-0.979)	<7.24	92.3 (66.7-99.6)	86.2 (75.1-92.8)	<2.52
**CSF p-tau_{217}**					
>0	0.799 (0.680-0.917)	>9.50	47.8 (34.1-61.9)	100 (79.6-100)	>8.95
>8	0.838 (0.741-0.935)	>4.90	84.4 (68.2-93.1)	65.5 (47.3-80.1)	>13.85
>16	0.886 (0.805-0.967)	>8.95	74.1 (55.3-86.8)	88.2 (73.4-95.3)	>13.85
>24	0.901 (0.824-0.978)	>8.95	85.0 (64.0-94.8)	82.9 (68.7-91.5)	>14.15
>32	0.941 (0.868-1.000)	>9.65	93.3 (70.2-99.7)	84.8 (71.8-92.4)	>14.15
>40	0.953 (0.901-1.000)	>9.65	100 (74.1-100)	80.0 (67.0-88.8)	>17.05
**CSF $A\beta_{42}$/p-tau_{217}**					
>0	0.852 (0.756-0.949)	<129.3	84.8 (71.8-92.4)	80.0 (54.8-92.9)	<63.97
>8	0.888 (0.810-0.966)	<52.90	68.7 (51.4-82.0)	93.1 (78.0-98.8)	<52.90
>16	0.934 (0.877-0.990)	<51.69	74.1 (55.3-86.8)	97.1 (85.1-99.8)	<52.27
>24	0.971 (0.953-1.000)	<48.35	90.0 (69.9-98.2)	95.1 (83.9-99.1)	<48.35
>32	0.980 (0.952-1.000)	<44.94	100 (79.6-100)	91.3 (79.7-96.6)	<31.96
>40	0.956 (0.908-1.000)	<44.94	100 (74.1-100)	84.0 (71.5-91.7)	<22.16
**Plasma cohort (n=101)**					
**Plasma $A\beta_{42}/A\beta_{40}$**					
>0	0.603 (0.489-0.718)	<0.076	44.9 (34.3-55.9)	87.0 (67.9-95.5)	<0.068
>8	0.628 (0.518-0.738)	<0.076	51.7 (39.3-63.8)	82.9 (68.7-91.5)	<0.065
>16	0.655 (0.543-0.766)	<0.076	57.1 (43.3-70.0)	80.8 (68.1-89.2)	<0.065
>24	0.600 (0.477-0.722)	<0.075	55.0 (39.8-69.3)	77.0 (65.1-85.8)	<0.063
>32	0.529 (0.385-0.674)	<0.073	46.4 (29.5-64.2)	76.7 (65.8-84.9)	<0.062
>40	0.556 (0.395-0.718)	<0.073	52.6 (31.7-72.7)	75.6 (65.3-83.6)	<0.060

**Non-prion plasma cohort (n=60)**					
**Plasma $A\beta_{42}/A\beta_{40}$**					
>0	0.635 (0.490-0.780)	<0.079	59.6 (45.3-72.4)	76.9 (49.7-91.8)	<0.072
>8	0.565 (0.418-0.713)	<0.076	51.3 (35.9-66.5)	78.3 (58.1-90.3)	<0.063
>16	0.613 (0.466-0.759)	<0.076	56.2 (39.3-71.8)	78.6 (60.5-89.8)	<0.063
>24	0.580 (0.421-0.739)	<0.073	50.0 (32.1-67.9)	79.4 (63.2-89.6)	<0.063
>32	0.543 (0.369-0.718)	<0.073	52.4 (32.4-71.7)	76.9 (61.7-87.3)	<0.063
>40	0.611 (0.418-0.804)	<0.073	64.3 (38.8-83.7)	76.1 (62.1-86.1)	<0.060
**Plasma p-tau$_{217}$**					
>0	0.719 (0.575-0.864)	>0.359	42.5 (29.5-56.7)	100 (77.2-100)	>0.340
>8	0.761 (0.639-0.882)	>0.213	81.1 (65.8-90.5)	60.9 (40.8-77.8)	>0.531
>16	0.802 (0.693-0.911)	>0.267	75.0 (57.9-86.7)	75.0 (55.6-87.3)	>0.531
>24	0.789 (0.671-0.907)	>0.379	61.5 (42.5-77.6)	91.2 (77.0-96.9)	>0.581
>32	0.902 (0.823-0.981)	>0.379	76.2 (54.9-89.4)	92.3 (79.7-97.3)	>0.629
>40	0.840 (0.724-0.956)	>0.379	78.6 (52.4-92.4)	82.6 (69.3-90.9)	>0.808
**Plasma p-tau$_{217}$/$A\beta_{42}$**					
>0	0.731 (0.567-0.894)	>0.014	55.3 (41.2-68.6)	84.6 (57.8-97.3)	>0.024
>8	0.759 (0.628-0.890)	>0.008	83.8 (68.9-92.3)	60.9 (40.8-77.8)	>0.026
>16	0.815 (0.705-0.926)	>0.014	71.9 (54.6-84.4)	82.1 (64.4-92.1)	>0.026
>24	0.806 (0.698-0.914)	>0.011	80.8 (62.1-91.5)	67.6 (50.8-80.9)	>0.026
>32	0.893 (0.815-0.970)	>0.014	90.5 (71.1-98.3)	76.9 (61.7-87.3)	>0.026
>40	0.856 (0.755-0.956)	>0.014	92.9 (68.5-99.6)	67.4 (53.0-79.1)	>0.034

Aβ score thresholds were defined using 8-unit intervals. Subjects with a score of zero were included in the lowest interval, whereas subjects with values above the 90th percentile of the entire cohort were grouped in the highest interval. Analyses on CSF p-tau$_{217}$ and $A\beta_{42}$/p-tau$_{217}$ were performed in a subgroup (n=61). AUC, sensitivity and specificity are expressed as value (95% confidence interval). Cut-off values for CSF p-tau$_{181}$ and p-tau$_{217}$ and plasma p-tau$_{217}$ are expressed in pg/ml. Abbreviations: AUC, area under the curve; CSF, cerebrospinal fluid

Supplementary Table 12 - Discriminatory performance of CSF and plasma biomarkers across increasing levels of tau pathology burden

	AUC	Maximized Youden-index cut-off			≃95% specificity cut-off
		Value	Sensitivity	Specificity	Value
**Non-prion CSF cohort (n=71)**					
**CSF p-tau$_{181}$**					
>0	0.664 (0.528-0.799)	>46.5	45.4 (33.0-58.5)	87.5 (64.0-97.8)	>79.0
>5	0.795 (0.688-0.902)	>46.5	68.7 (51.4-82.0)	87.2 (73.3-94.4)	>79.0
>10	0.900 (0.819-0.977)	>37.5	95.2 (77.3-99.8)	74.0 (60.4-84.1)	>79.0
>15	0.949 (0.902-0.996)	>46.5	100 (81.6-100)	81.5 (69.2-89.6)	>79.0
>20	0.902 (0.832-0.973)	>53.0	100 (77.2-100)	77.6 (65.3-86.4)	>115.5
>25	0.855 (0.766-0.945)	>53.0	100 (67.6-100)	71.4 (59.3-81.1)	>150.5
**CSF Aβ$_{42}$/p-tau$_{181}$**					
>0	0.818 (0.715-0.922)	<13.87	76.4 (63.6-85.6)	81.2 (57.0-93.4)	<11.19
>5	0.866 (0.779-0.953)	<7.68	62.5 (45.2-77.1)	97.4 (86.8-99.9)	<7.99
>10	0.952 (0.899-1.000)	<7.24	85.7 (65.4-95.0)	96.0 (86.5-99.3)	<7.68
>15	0.992 (0.979-1.000)	<7.24	100 (81.6-100)	94.4 (84.9-98.5)	<6.68
>20	0.971 (0.937-1.000)	<5.98	100 (77.2-100)	93.1 (83.6-97.3)	<3.20
>25	0.950 (0.896-1.000)	<5.98	100 (67.6-100)	85.7 (75.0-92.3)	<2.57
**CSF p-tau$_{217}$**					
>0	0.691 (0.557-0.826)	>9.50	46.8 (33.3-60.8)	100 (78.5-100)	>9.30
>5	0.853 (0.754-0.953)	>9.50	70.4 (51.5-84.1)	91.2 (77.0-96.9)	>11.25
>10	0.961 (0.917-1.000)	>9.50	94.1 (73.0-99.7)	86.4 (73.3-93.6)	>13.10
>15	0.994 (0.979-1.000)	>15.25	92.3 (66.7-99.6)	100 (92.6-100)	>13.85
>20	0.969 (0.923-1.000)	>17.05	100 (70.1-100)	96.1 (87.0-99.3)	>15.25
>25	0.934 (0.863-1.000)	>17.05	100 (51.0-100)	87.7 (76.7-93.9)	>50.90
**CSF Aβ$_{42}$/p-tau$_{217}$**					
>0	0.801 (0.679-0.922)	<62.77	61.7 (47.4-74.2)	92.9 (68.5-99.6)	<62.77
>5	0.893 (0.809-0.978)	<62.77	88.9 (71.9-96.1)	82.3 (66.5-91.6)	<48.35
>10	0.979 (0.949-1.000)	<48.35	94.1 (73.0-99.7)	90.9 (78.8-96.4)	<43.41
>15	0.995 (0.984-1.000)	<31.96	100 (77.2-100)	97.9 (89.1-99.9)	<39.02
>20	0.972 (0.934-1.000)	<22.16	100 (70.1-100)	94.2 (84.4-98.4)	<22.16
>25	0.930 (0.859-1.000)	<22.16	100 (51.1-100)	86.0 (74.7-92.7)	<6.77
**Non-prion plasma cohort (n=60)**					
**Plasma p-tau$_{217}$**					
>0	0.728 (0.594-0.863)	>0.299	55.1 (41.3-68.1)	100 (74.1-100)	>0.277
>5	0.797 (0.685-0.909)	>0.288	73.3 (55.5-85.8)	76.7 (59.1-88.2)	>0.670
>10	0.821 (0.710-0.931)	>0.359	68.2 (47.3-83.6)	86.8 (72.7-94.2)	>0.693
>15	0.879 (0.794-0.963)	>0.359	75.0 (53.1-88.8)	87.5 (73.9-94.5)	>0.693
>20	0.861 (0.765-0.956)	>0.288	93.3 (70.2-99.7)	66.7 (52.1-78.6)	>0.808
>25	0.912 (0.827-0.997)	>0.531	88.9 (56.5-99.4)	88.2 (76.6-94.5)	>0.886
**Plasma p-tau$_{217}$/Aβ$_{42}$**					
>0	0.740 (0.584-0.897)	>0.011	61.2 (47.2-73.6)	81.8 (52.3-96.7)	>0.021
>5	0.819 (0.713-0.925)	>0.011	80.0 (62.7-90.5)	73.3 (55.5-85.8)	>0.024
>10	0.877 (0.790-0.965)	>0.014	86.4 (66.7-95.2)	76.3 (60.8-87.0)	>0.024
>15	0.919 (0.852-0.985)	>0.014	95.0 (76.4-99.7)	77.5 (62.5-87.7)	>0.026
>20	0.921 (0.853-0.988)	>0.014	100 (79.6-100)	71.1 (56.6-82.3)	>0.026
>25	0.928 (0.848-1.000)	>0.021	88.9 (56.5-99.4)	82.3 (69.7-90.4)	>0.034

Tau score thresholds were defined using 5-unit intervals. Subjects with a score of zero were included in the lowest interval, whereas subjects with values above the 90th percentile of the entire cohort were grouped in the highest interval. Analyses on CSF p-tau$_{217}$ and Aβ$_{42}$/p-tau$_{217}$ were performed in a subgroup (n=61). AUC, sensitivity and specificity are expressed as value (95% confidence interval). Cut-off values for CSF p-tau$_{181}$ and p-tau$_{217}$ and plasma p-tau$_{217}$ are expressed in pg/ml. Abbreviations: AUC, area under the curve; CSF, cerebrospinal fluid

Supplementary Table 13 - Discriminatory performance of CSF and plasma biomarkers across increasing Thal phases

	AUC	Maximized Youden-index cut-off			≃95% specificity cut-off
		Value	Sensitivity	Specificity	Value
**CSF cohort (n=230)**					
**CSF Aβ42/Aβ40**					
>0	0.829 (0.777-0.882)	<0.080	70.3 (62.7-76.9)	84.0 (74.1-90.6)	<0.074
>1	0.862 (0.814-0.911)	<0.073	65.5 (56.6-73.5)	94.6 (88.7-97.5)	<0.073
>2	0.925 (0.891-0.959)	<0.070	77.6 (67.7-85.2)	93.8 (88.6-96.7)	<0.069
>3	0.951 (0.924-0.978)	<0.066	95.0 (83.5-99.1)	86.8 (81.3-90.9)	<0.051
>4	0.915 (0.874-0.957)	<0.055	91.3 (73.2-98.4)	87.4 (82.2-91.3)	<0.044
**Non-prion CSF cohort (n=71)**					
**CSF Aβ42/Aβ40**					
>0	0.840 (0.746-0.933)	<0.076	73.1 (59.7-83.2)	94.7 (75.4-99.7)	<0.076
>1	0.916 (0.845-0.987)	<0.076	86.0 (72.7-93.4)	92.9 (77.3-98.7)	<0.074
>2	0.976 (0.948-1.000)	<0.070	91.2 (77.0-96.9)	94.6 (82.3-99.0)	<0.070
>3	0.957 (0.915-0.999)	<0.054	94.4 (74.2-99.7)	86.8 (75.2-93.4)	<0.047
>4	0.911 (0.844-0.978)	<0.054	100 (75.7-100)	79.7 (67.7-88.0)	<0.041
**CSF p-tau181**					
>0	0.701 (0.566-0.835)	>25.5	78.8 (66.0-87.8)	57.9 (36.4-76.9)	>79.0
>1	0.766 (0.650-0.881)	>25.5	88.4 (75.5-94.9)	60.7 (42.4-76.4)	>81.0
>2	0.809 (0.708-0.910)	>37.5	76.5 (60.0-87.6)	81.1 (65.8-90.5)	>81.0
>3	0.872 (0.787-0.956)	>37.5	94.4 (74.2-99.7)	69.8 (56.5-80.5)	>93.5
>4	0.877 (0.791-0.964)	>46.5	91.7 (64.6-99.6)	72.9 (60.4-82.6)	>115.5
**CSF Aβ42/p-tau181**					
>0	0.757 (0.641-0.873)	<13.36	73.1 (59.7-83.2)	73.7 (51.2-88.2)	<9.31
>1	0.797 (0.694-0.900)	<14.91	83.7 (70.0-91.9)	64.3 (45.8-79.3)	<7.99
>2	0.882 (0.808-0.956)	<9.03	67.6 (50.8-80.9)	91.9 (78.7-97.2)	<7.99
>3	0.922 (0.852-0.993)	<7.99	88.9 (67.2-98.0)	88.7 (77.4-94.7)	<5.98
>4	0.907 (0.827-0.986)	<7.24	91.7 (64.6-99.6)	84.7 (73.5-91.8)	<2.57
**CSF p-tau217**					
>0	0.784 (0.661-0.907)	>9.50	47.7 (33.7-62.1)	94.1 (73.0-99.7)	>9.50
>1	0.844 (0.747-0.941)	>3.30	94.4 (81.9-99.0)	56.0 (37.1-73.3)	>13.10
>2	0.861 (0.772-0.950)	>8.95	69.0 (50.8-82.7)	87.5 (71.9-95.0)	>13.10
>3	0.952 (0.899-1.000)	>8.95	100 (77.2-100)	77.1 (63.5-86.7)	>15.25
>4	0.934 (0.859-1.000)	>9.65	100 (67.6-100)	75.5 (62.4-85.1)	>39.65
**CSF Aβ42/p-tau217**					
>0	0.822 (0.718-0.926)	<129.30	84.1 (70.6-92.1)	70.6 (46.9-86.7)	<56.49
>1	0.874 (0.787-0.962)	<129.30	94.4 (81.9-99.0)	68.0 (48.4-82.8)	<52.90
>2	0.908 (0.839-0.977)	<51.69	69.0 (50.8-82.7)	96.9 (84.3-99.8)	<52.27
>3	0.968 (0.928-1.000)	<44.94	100 (77.2-100)	87.5 (75.3-94.1)	<22.16
>4	0.915 (0.843-0.988)	<44.94	100 (67.6-100)	79.2 (66.5-88.0)	<8.26
**Plasma cohort (n=101)**					
**Plasma Aβ42/Aβ40**					
>0	0.595 (0.481-0.709)	<0.076	45.4 (34.8-56.5)	87.5 (69.0-95.7)	<0.068
>1	0.616 (0.505-0.728)	<0.076	48.4 (36.6-60.4)	81.1 (65.8-90.5)	<0.063
>2	0.642 (0.531-0.753)	<0.076	56.0 (42.3-68.8)	80.4 (67.5-89.0)	<0.063
>3	0.637 (0.506-0.769)	<0.075	61.3 (43.8-76.3)	75.7 (64.5-84.2)	<0.063
>4	0.563 (0.406-0.719)	<0.073	52.6 (31.7-72.7)	75.6 (65.3-83.6)	<0.060
**Non-prion plasma cohort (n=60)**					
**Plasma Aβ42/Aβ40**					
>0	0.621 (0.478-0.764)	<0.076	47.8 (34.1-61.9)	85.7 (60.1-97.5)	<0.072
>1	0.561 (0.409-0.712)	<0.079	60.0 (44.6-73.6)	65.0 (43.3-81.9)	<0.063
>2	0.615 (0.469-0.762)	<0.075	56.2 (39.3-71.8)	82.1 (64.4-92.1)	<0.063
>3	0.630 (0.464-0.796)	<0.073	57.1 (36.5-75.5)	79.5 (64.5-89.2)	<0.063
>4	0.592 (0.410-0.773)	<0.073	57.1 (32.6-78.6)	73.9 (59.7-84.4)	<0.056

**Plasma p-tau$_{217}$**					
>0	0.724 (0.587-0.862)	>0.359	43.5 (30.2-57.7)	100 (78.5-100)	>0.340
>1	0.761 (0.639-0.884)	>0.359	47.5 (32.9-62.5)	95.0 (76.4-99.7)	>0.359
>2	0.793 (0.682-0.904)	>0.359	56.2 (39.3-71.8)	92.9 (77.3-98.7)	>0.531
>3	0.837 (0.733-0.941)	>0.379	66.7 (45.4-82.8)	87.2 (73.3-94.4)	>0.670
>4	0.824 (0.704-0.944)	>0.379	71.4 (45.3-88.3)	80.4 (66.8-89.3)	>0.808
**Plasma p-tau$_{217}$/Aβ$_{42}$**					
>0	0.742 (0.588-0.896)	>0.014	56.5 (42.2-69.8)	85.7 (60.1-97.5)	>0.024
>1	0.780 (0.649-0.910)	>0.014	62.5 (47.0-75.8)	85.0 (64.0-94.8)	>0.026
>2	0.801 (0.688-0.914)	>0.014	68.7 (51.4-82.0)	78.6 (60.5-89.8)	>0.026
>3	0.842 (0.746-0.939)	>0.011	90.5 (71.1-98.3)	66.7 (51.0-79.4)	>0.028
>4	0.807 (0.696-0.919)	>0.011	92.9 (68.5-99.6)	58.7 (44.3-71.7)	>0.043

Analyses on CSF p-tau$_{217}$ and Aβ$_{42}$/p-tau$_{217}$ were performed in a subgroup (n=61). AUC, sensitivity and specificity are expressed as value (95% confidence interval). Cut-off values for CSF p-tau$_{181}$ and p-tau$_{217}$ and plasma p-tau$_{217}$ are expressed in pg/ml. Abbreviations: AUC, area under the curve; CSF, cerebrospinal fluid

Supplementary Table 14 - Discriminatory performance of CSF and plasma biomarkers across increasing Braak stages

	AUC	Maximized Youden-index cut-off			≃95% specificity cut-off
		Value	Sensitivity	Specificity	Value
**Non-prion CSF cohort (n=71)**					
**CSF p-tau$_{181}$**					
>0	0.762 (0.653-0.872)	>37.5	65.1 (50.2-77.6)	82.1 (64.4-92.1)	>79.0
>1	0.814 (0.701-0.926)	>46.5	74.1 (55.3-86.8)	84.1 (70.6-92.1)	>79.0
>2	0.913 (0.830-0.995)	>46.5	94.4 (74.2-99.7)	81.1 (68.6-89.4)	>79.0
>3	0.892 (0.817-0.967)	>53.0	100 (75.7-100)	76.3 (64.0-85.3)	>147.5
>4	0.894 (0.814-0.974)	>81.0	100 (56.5-100)	84.8 (74.3-91.6)	>150.5
>5	0.986 (0.958-1.000)	>178.0	100 (5.1-100)	98.6 (92.3-99.9)	>150.5
**CSF Aβ_{42}/p-tau$_{181}$**					
>0	0.839 (0.747-0.930)	<13.36	83.7 (70.0-91.9)	75.0 (56.6-87.3)	<7.99
>1	0.889 (0.808-0.970)	<7.24	70.4 (51.5-84.1)	97.7 (88.2-99.9)	<7.68
>2	0.969 (0.920-1.000)	<7.24	94.4 (74.2-99.7)	94.3 (84.6-98.5)	<7.24
>3	0.956 (0.911-1.000)	<5.98	100 (75.7-100)	91.5 (81.6-96.3)	<3.20
>4	0.945 (0.880-1.000)	<5.23	100 (56.5-100)	83.3 (72.6-90.4)	<2.49
>5	0.957 (0.910-1.000)	<1.94	100 (5.1-100)	95.7 (88.1-98.8)	<2.14
**CSF p-tau$_{217}$**					
>0	0.804 (0.697-0.911)	>9.50	56.8 (40.9-71.3)	95.8 (79.8-99.8)	>9.50
>1	0.871 (0.763-0.980)	>9.50	81.8 (61.5-92.7)	89.7 (76.4-95.9)	>11.60
>2	0.971 (0.924-1.000)	>15.25	85.7 (60.1-97.5)	100 (92.4-100)	>13.85
>3	0.965 (0.920-1.000)	>17.05	100 (67.6-100)	94.3 (84.6-98.5)	>17.05
>4	0.983 (0.950-1.000)	>55.80	100 (17.8-100)	98.3 (91.0-99.9)	>50.90
>5	na	na	na	na	na
**CSF Aβ_{42}/p-tau$_{217}$**					
>0	0.855 (0.761-0.948)	<93.88	83.9 (68.9-92.3)	70.8 (50.8-85.1)	<48.35
>1	0.908 (0.828-0.988)	<54.70	86.4 (66.7-95.2)	84.6 (70.3-92.7)	<43.41
>2	0.986 (0.963-1.000)	<31.96	92.9 (68.5-99.6)	97.9 (88.9-99.9)	<39.02
>3	0.965 (0.920-1.000)	<22.16	100 (67.6-100)	92.4 (82.1-97.0)	<12.74
>4	0.949 (0.880-1.000)	<8.26	100 (17.8-100)	91.5 (81.6-96.3)	<6.03
>5	na	na	na	na	na
**Non-prion plasma cohort (n=60)**					
**Plasma p-tau$_{217}$**					
>0	0.748 (0.618-0.878)	>0.299	58.5 (43.4-72.2)	84.2 (62.4-94.5)	>0.359
>1	0.782 (0.666-0.899)	>0.359	61.5 (42.5-77.5)	88.2 (73.4-95.3)	>0.693
>2	0.842 (0.737-0.948)	>0.359	71.4 (50.0-86.2)	87.2 (73.3-94.4)	>0.693
>3	0.908 (0.831-0.984)	>0.288	100 (78.5-100)	67.4 (53.0-79.1)	>0.736
>4	0.912 (0.820-1.000)	>0.531	87.5 (52.9-99.3)	86.5 (74.7-93.3)	>0.808
>5	0.767 (0.480-1.000)	>0.299	100 (17.8-100)	56.9 (44.1-68.8)	>1.031
**Plasma p-tau$_{217}$/Aβ_{42}**					
>0	0.819 (0.704-0.934)	>0.011	70.7 (55.5-82.4)	84.2 (62.4-94.5)	>0.021
>1	0.817 (0.712-0.922)	>0.011	80.8 (62.1-91.5)	67.6 (50.8-80.9)	>0.024
>2	0.891 (0.807-0.975)	>0.014	90.5 (71.1-98.3)	76.9 (61.7-87.3)	>0.024
>3	0.948 (0.894-1.000)	>0.017	92.9 (68.5-99.6)	84.8 (71.8-92.4)	>0.026
>4	0.939 (0.855-1.000)	>0.028	87.5 (52.9-99.4)	92.3 (81.8-97.0)	>0.034
>5	0.724 (0.507-0.941)	>0.014	100 (17.8-100)	55.2 (42.4-67.2)	>0.050

Analyses on CSF p-tau$_{217}$ and Aβ_{42}/p-tau$_{217}$ were performed in a subgroup (n=61). AUC, sensitivity and specificity are expressed as value (95% confidence interval). Cut-off values for CSF p-tau$_{181}$ and p-tau$_{217}$ and plasma p-tau$_{217}$ are expressed in pg/ml. Abbreviations: AUC, area under the curve; CSF, cerebrospinal fluid; na, not available

Supplementary Table 15 - Discriminatory performance of CSF and plasma biomarkers across increasing ADNC levels

	AUC	Maximized Youden-index cut-off			≃95% specificity cut-off
		Value	Sensitivity	Specificity	Value
**CSF cohort (n=230)**					
**CSF $A\beta_{42}/A\beta_{40}$**					
>Not AD	0.829 (0.777-0.882)	<0.080	70.3 (62.7-76.9)	84.0 (74.1-90.6)	<0.074
>Low	0.938 (0.897-0.980)	<0.058	93.9 (80.4-98.9)	88.3 (83.1-92.1)	<0.049
>Int.	0.902 (0.843-0.960)	<0.054	100 (56.5-100)	82.2 (76.7-86.7)	<0.037
**Non-prion CSF cohort (n=71)**					
**CSF $A\beta_{42}/A\beta_{40}$**					
>Not AD	0.840 (0.746-0.933)	<0.076	73.1 (59.7-83.2)	94.7 (75.4-99.7)	<0.076
>Low	0.971 (0.938-1.000)	<0.054	100 (82.4-100)	88.7 (77.4-94.7)	<0.047
>Int.	0.838 (0.730-0.946)	<0.054	100 (56.5-100)	71.2 (59.4-80.7)	<0.031
**CSF p-tau$_{181}$**					
>Not AD	0.701 (0.566-0.835)	>25.5	78.8 (66.0-87.8)	57.9 (36.3-76.9)	>79.0
>Low	0.913 (0.830-0.995)	>46.5	94.4 (74.2-99.7)	81.1 (68.6-89.4)	>79.0
>Int.	0.894 (0.814-0.974)	>81.0	100 (56.5-100)	84.8 (74.3-91.6)	>150.5
**CSF $A\beta_{42}$/p-tau$_{181}$**					
>Not AD	0.757 (0.641-0.873)	<13.36	73.1 (59.7-83.2)	73.7 (51.2-88.2)	<9.31
>Low	0.969 (0.920-1.000)	<7.24	94.4 (74.2-99.7)	94.3 (84.6-98.5)	<7.24
>Int.	0.945 (0.880-1.000)	<5.23	100 (56.5-100)	83.3 (72.6-90.4)	<2.49
**CSF p-tau$_{217}$**					
>Not AD	0.784 (0.661-0.907)	>9.50	47.7 (33.7-62.1)	94.1 (73.0-99.7)	>9.50
>Low	0.971 (0.924-1.000)	>15.25	85.7 (60.1-97.5)	100 (92.4-100)	>13.85
>Int.	0.983 (0.950-1.000)	>55.80	100 (17.8-100)	98.3 (91.0-99.9)	>50.90
**CSF $A\beta_{42}$/p-tau$_{217}$**					
>Not AD	0.822 (0.718-0.926)	<129.30	84.1 (70.6-92.1)	70.6 (46.9-86.7)	<56.49
>Low	0.986 (0.963-1.000)	<31.96	92.9 (68.5-99.6)	97.9 (88.9-99.9)	<39.02
>Int.	0.949 (0.880-1.000)	<8.26	100 (17.7-100)	91.5 (81.6-96.3)	<6.03
**Plasma cohort (n=101)**					
**Plasma $A\beta_{42}/A\beta_{40}$**					
>Not AD	0.595 (0.481-0.709)	<0.076	45.4 (34.8-56.5)	87.5 (69.0-95.7)	<0.068
>Low	0.561 (0.422-0.700)	<0.073	50.0 (32.6-67.4)	78.1 (67.3-86.0)	<0.062
>Int.	0.571 (0.318-0.824)	>0.096	37.5 (13.7-69.4)	90.3 (82.6-94.8)	>0.108
**Non-prion plasma cohort (n=60)**					
**Plasma $A\beta_{42}/A\beta_{40}$**					
>Not AD	0.621 (0.478-0.764)	<0.076	47.8 (34.1-61.9)	85.7 (60.1-97.5)	<0.072
>Low	0.522 (0.351-0.694)	<0.073	50.0 (29.9-70.1)	75.0 (59.8-85.8)	<0.063
>Int.	0.594 (0.346-0.841)	>0.095	37.5 (13.7-69.4)	92.3 (81.8-97.0)	>0.112
**Plasma p-tau$_{217}$**					
>Not AD	0.724 (0.587-0.862)	>0.359	43.5 (30.2-57.7)	100 (78.5-100)	>0.340
>Low	0.831 (0.722-0.941)	>0.288	85.0 (64.0-94.8)	70.0 (54.6-81.9)	>0.693
>Int.	0.912 (0.820-1.000)	>0.531	87.5 (52.9-99.4)	86.5 (74.7-93.3)	>0.808
**Plasma p-tau$_{217}$/$A\beta_{42}$**					
>Not AD	0.742 (0.588-0.896)	>0.014	56.5 (42.2-69.8)	85.7 (60.1-97.5)	>0.024
>Low	0.879 (0.790-0.967)	>0.014	90.0 (69.9-98.2)	75.0 (59.8-85.8)	>0.026
>Int.	0.939 (0.855-1.000)	>0.028	87.5 (52.9-99.4)	92.3 (81.8-97.0)	>0.034

Analyses on CSF p-tau$_{217}$ and $A\beta_{42}$/p-tau$_{217}$ were performed in a subgroup (n=61). AUC, sensitivity and specificity are expressed as value (95% confidence interval). Cut-off values for CSF p-tau$_{181}$ and p-tau$_{217}$ and plasma p-tau$_{217}$ are expressed in pg/ml. Abbreviations: AD, Alzheimer's disease; ADNC, Alzheimer's disease neuropathologic change; AUC, area under the curve; CSF, cerebrospinal fluid; Int, intermediate

SUPPLEMENTARY FIGURES

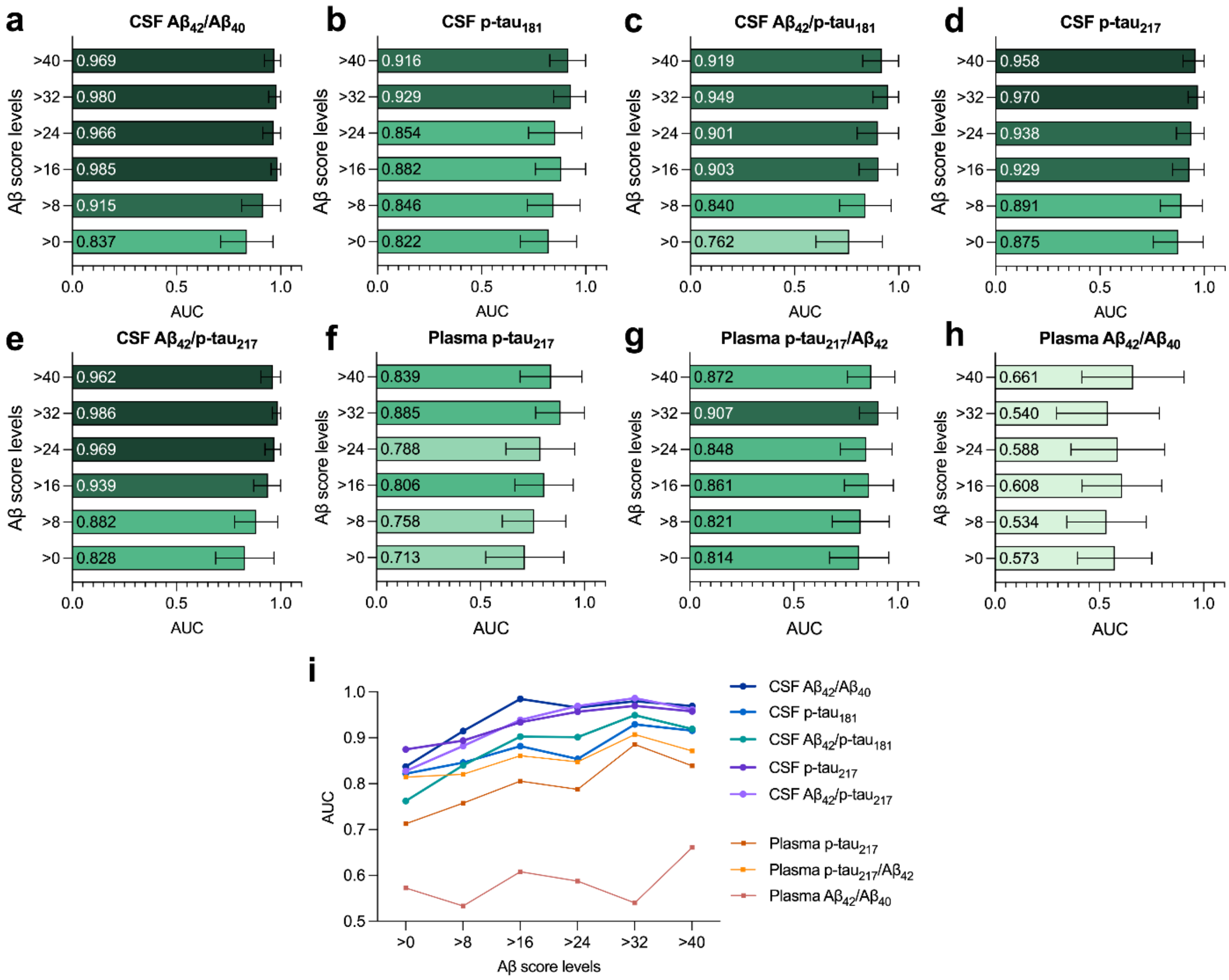

Supplementary Fig. 1 Discriminatory performance of biofluid biomarkers across increasing Aβ pathology burden in the subgroup of non-prion participants with complete biomarker data available (n=38). a-h: Bar plots of AUC values for each biomarker for discriminating progressively higher Aβ burden, defined by increasing Aβ score thresholds (y-axis). Error bars represent 95% confidence intervals. Increasing shades of green reflect higher AUC values. **i**: Line plot showing AUC values across Aβ score thresholds for all biomarkers. 95% confidence intervals are not shown for graphical reasons. Abbreviations: AUC, area under the curve

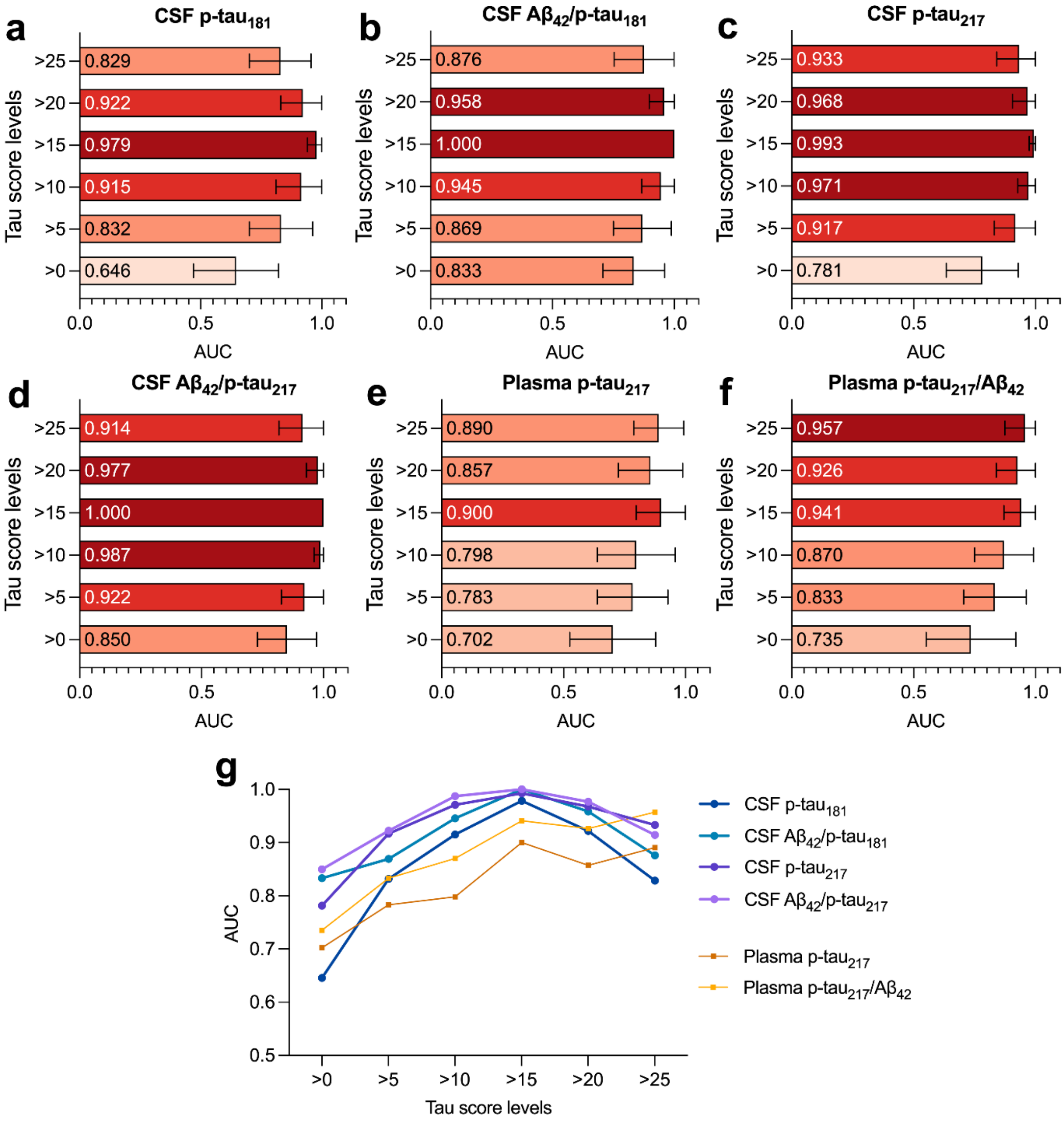

Supplementary Fig. 2 Discriminatory performance of biofluid biomarkers across increasing tau pathology burden in the subgroup of non-prion participants with complete biomarker data available (n=38). a-f: Bar plots of AUC values for each biomarker for discriminating progressively higher tau burden, defined by increasing tau score thresholds (y-axis). Error bars represent 95% confidence intervals. Increasing shades of red reflect higher AUC values. **g**: Line plot showing AUC values across tau score thresholds for all biomarkers. 95% confidence intervals are not shown for graphical reasons. Abbreviations: AUC, area under the curve
